# Cell-Surface Marker Signatures for the Isolation of Neural Stem Cells, Glia and Neurons Derived from Human Pluripotent Stem Cells

**DOI:** 10.1371/journal.pone.0017540

**Published:** 2011-03-02

**Authors:** Shauna H. Yuan, Jody Martin, Jeanne Elia, Jessica Flippin, Rosanto I. Paramban, Mike P. Hefferan, Jason G. Vidal, Yangling Mu, Rhiannon L. Killian, Mason A. Israel, Nil Emre, Silvia Marsala, Martin Marsala, Fred H. Gage, Lawrence S. B. Goldstein, Christian T. Carson

**Affiliations:** 1 Howard Hughes Medical Institute and Department of Cellular and Molecular Medicine, School of Medicine, University of California San Diego, La Jolla, California, United States of America; 2 Department of Neurosciences, School of Medicine, University of California San Diego, La Jolla, California, United States of America; 3 BD Biosciences, La Jolla, California, United States of America; 4 Anesthesiology Research Laboratory, Department of Anesthesiology, University of California San Diego, La Jolla, California, United States of America; 5 Laboratory of Genetics, The Salk Institute for Biological Studies, La Jolla, California, United States of America; 6 Biomedical Sciences Graduate Program, University of California San Diego, La Jolla, California, United States of America; 7 Institute of Neurobiology, Slovak Academy of Sciences, Košice, Slovakia; University of Southern California, United States of America

## Abstract

**Background:**

Neural induction of human pluripotent stem cells often yields heterogeneous cell populations that can hamper quantitative and comparative analyses. There is a need for improved differentiation and enrichment procedures that generate highly pure populations of neural stem cells (NSC), glia and neurons. One way to address this problem is to identify cell-surface signatures that enable the isolation of these cell types from heterogeneous cell populations by fluorescence activated cell sorting (FACS).

**Methodology/Principal Findings:**

We performed an unbiased FACS- and image-based immunophenotyping analysis using 190 antibodies to cell surface markers on naïve human embryonic stem cells (hESC) and cell derivatives from neural differentiation cultures. From this analysis we identified prospective cell surface signatures for the isolation of NSC, glia and neurons. We isolated a population of NSC that was CD184^+^/CD271^−^/CD44^−^/CD24^+^ from neural induction cultures of hESC and human induced pluripotent stem cells (hiPSC). Sorted NSC could be propagated for many passages and could differentiate to mixed cultures of neurons and glia in vitro and in vivo. A population of neurons that was CD184^−^/CD44^−^/CD15^LOW^/CD24^+^ and a population of glia that was CD184^+^/CD44^+^ were subsequently purified from cultures of differentiating NSC. Purified neurons were viable, expressed mature and subtype-specific neuronal markers, and could fire action potentials. Purified glia were mitotic and could mature to GFAP-expressing astrocytes in vitro and in vivo.

**Conclusions/Significance:**

These findings illustrate the utility of immunophenotyping screens for the identification of cell surface signatures of neural cells derived from human pluripotent stem cells. These signatures can be used for isolating highly pure populations of viable NSC, glia and neurons by FACS. The methods described here will enable downstream studies that require consistent and defined neural cell populations.

## Introduction

Human embryonic stem cells (hESC) and human induced pluripotent stem cells (hiPSC) have the ability to differentiate to somatic-like cells [1], [2], [3]. Thus, hESC and hiPSC differentiation offers a unique opportunity for therapy development, drug screening, disease modeling, and tissue replacement. However, developing well-defined conditions to generate pure populations of specific cell types is critical to achieve these goals.

There are several neural induction methods that enrich for NSC or neurons using spontaneous differentiation, chemical induction or mouse stromal feeder cells [4], [5], [6], [7], [8], [9]. NSC can be manually isolated and be propagated as monolayer cultures for many passages [10], [11]. In principle, these cells can differentiate to neurons and glia, providing an endless supply of cells for in vitro and in vivo assays. Unfortunately, the robustness of these methods is hampered by batch-to-batch variability of isolated NSC. Moreover, differentiation of NSC often results in variable and heterogeneous cultures of neurons, glia and undifferentiated cells, which impedes many downstream applications requiring purified or defined cell populations, such as in vitro assays, transplantation and microarrays [12], [13]. One possible solution to this problem is to identify cell surface markers expressed on NSC, glia and neurons to define and purify distinct cell types, similar to what has been accomplished in studies of hematopoiesis.

Cell surface marker expression has been described for the identification and isolation of many neural cell types by FACS from embryonic and adult tissue from multiple species. The glycoprotein CD133 is a known stem/progenitor cell marker in many tissues and has been used to isolate NSC from human brain [14], [15], [16], [17]. The carbohydrate moiety CD15, also known as stage-specific embryonic antigen-1 or LeX, has been used to isolate NSC and radial glia from the subventricular zone (SVZ) in mice [18], [19]. CD184, a G protein-coupled receptor, was successfully used in combination with CD15 to isolate NSC from mouse embryonic forebrain and adult SVZ [20]. CD24 is a cell adhesion molecule that has been used to isolate NSC from mouse brain by FACS [21], [22]. Maric et al. were successful in isolating neuronally-restricted cells and NSC from embryonic rat telencephalon based on surface expression of tetanus toxin fragment C and cholera toxin B subunit [23]. In addition, neural stem cells and neural progenitors have been isolated from human brain using genetic promoter-reporters of neural stem cell markers [24], [25].

Likewise, advancements have been made in the identification and isolation of hESC-derived neural cells by FACS. Pruszak et al. (2007) reported that cultures of hESC differentiating to neural lineages can be assayed at different developmental stages with cell surface markers and that neurons could be enriched using an antibody to CD56 (NCAM) [26]. CD184^+^/CD326^−^ have been used to purify neural progenitors capable of differentiation into neurons from differentiating hESC [27]. In addition, Peh et al. have reported the enrichment for neurosphere-forming NSC from neural induction cultures of hESC based on expression for CD133, CD15 and GCTM-2 [28]. Using a similar strategy, Golebiewska et al. used CD133^+^/CD45^−^/CD34^−^ to isolate NSC from differentiating hESC [29]. Pruszak et al. (2009) demonstrated the utility of CD24, CD15 and CD29 as a surface maker code for isolating distinct cell populations, including NSC and a mixed population of neuroblasts and neurons from neural induction cultures of hESC [30].

These studies have made significant progress, but there is still a need to develop improved methods for the purification of neural cell types. Here we aimed to identify cell surface signatures that would permit the isolation of NSC, glia and post-mitotic neurons from neural differentiation cultures of pluripotent stem cells by FACS. To this end, we performed an unbiased FACS- and image-based screen, utilizing 190 characterized antibodies to cell surface markers on multiple hESC-derived cell cultures. From this screen we identified a cell surface signature that was successful in enriching NSC from multiple common neural induction culture systems. In addition, we identified cell surface signatures for the isolation of neurons and glia from differentiating NSC cultures at very high purity. Furthermore, we validated these methods on a number of hESC and hiPSC lines.

## Results

### Antibody screens identify prospective markers for distinguishing NSC, neurons and glia

**Figure 1:**
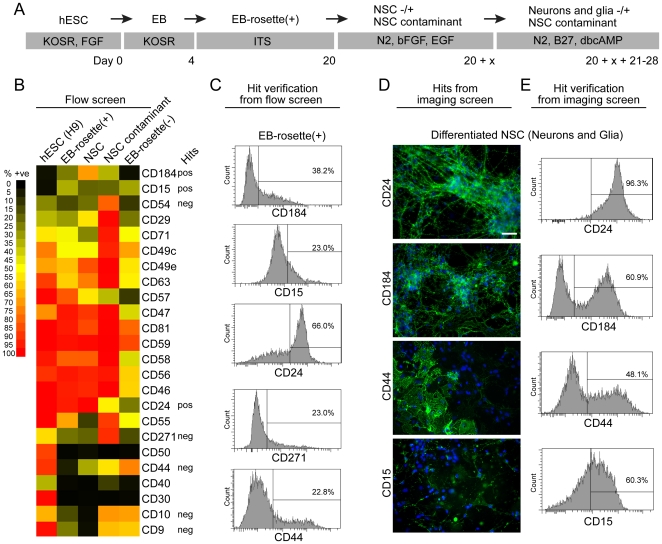
Cell surface marker screen of cell cultures at distinct phases of SFEB neural induction of H9 hESC. (A) Diagrammatic representation of the SFEB neural induction and isolation of NSC (see Methods for details). EB  =  embryoid body(ies); EB-rosette(+)  =  EB at a stage of differentiation when columnar rosettes are present in many EB; NSC  =  neuronal stem cells expanded from rosettes that were handpicked from EB-rosette(+); NSC contaminant  =  culture of intermittent contaminant of handpicked and expanded NSC cultures. (B) Heat map showing percent positive (% +ve) of representative cell surface markers in a FACS-based screen. EB-rosette(-)  =  EB depleted of rosettes. Markers identified from the screen to be potential positive (pos) or negative (neg) selection candidates (Hits) are noted. (C) Examples of intensity distributions of EB-rosette(+) cultures stained with candidate cell surface markers. The percentage reflects the population in the “positive” population for each marker. (D) Examples of an image screen of cell surface markers on neuron enriched cultures induced to differentiate from NSC for 3 weeks. CD24 clearly stains neurons whereas CD44, and CD15 do not. CD184 appears to stain some neurons and other cell types. (E) Same as c but with neuron-enriched cultures induced to differentiate from NSC. Scale bar is 50 µm.

We used the serum-free embryoid body (SFEB) culture method with the H9 hESC line to generate cell populations for a FACS- and image-based antibody screen (Fig. 1A). As previously reported SFEB robustly generated heterogeneous cultures containing neural ectoderm in the form of neural rosettes after plating EBs in adherent plates, referred to here as EB-rosette(+) [31], [32]. Neural rosettes exhibited Sox1 staining (Fig. S1A), suggesting that they were rich in NSC. Similar to previous results, we were able to manually isolate a population of NSC from EB-rosette(+) capable of subsequent expansion and differentiation to neurons and glia [11], [33]. Manually isolated NSC expressed neural stem cell markers Sox1, Pax6, Sox2 and Nestin, although Pax6 expression waned at later passages (Fig. S1B–E and data not shown). NSC could be induced to differentiate to mixed cultures of neurons, glia and undifferentiated NSC (Fig. S1F and data not shown). We observed that this method for generating NSC was not always successful, due to the inconsistent appearance of contaminating cells that outgrew NSC in many cultures (NSC contaminants) (Fig. S1H–J). Similar cell contaminants have been observed by others (Alysson Muotri and Mark Tomishima, personal communications).

We performed an unbiased FACS- and image-based screen using a collection of 190 characterized monoclonal antibodies to cell surface markers on cell cultures at distinct phases of neural induction by SFEB: naïve hESC, EB-rosette(+), manually picked and expanded NSC, and NSC that had been differentiated for 3 weeks to mixed populations of neurons and glia (Fig. 1, Fig. S2). To identify markers of potentially contaminating cell types, we screened a cell culture enriched in the NSC contaminant that overgrew during a preparation of NSC and a cell culture of EB that were depleted of neural ectoderm (EB-rosette(-)) (Fig. S1K). We screened all of the cell populations by FACS except for the differentiated NSC, which we screened by image to take advantage of the distinct morphological differences between neurons and non-neuronal cells. We co-stained these cultures with anti-β-III tubulin to identify neurons (data not shown). “Hits” identified from the screens were verified by FACS (Fig. 1C, E).

### CD184^+^/CD271^−^/CD44^−^/CD24^+^ enable the isolation of NSC from neural induction cultures of pluripotent stem cells

**Figure 2:**
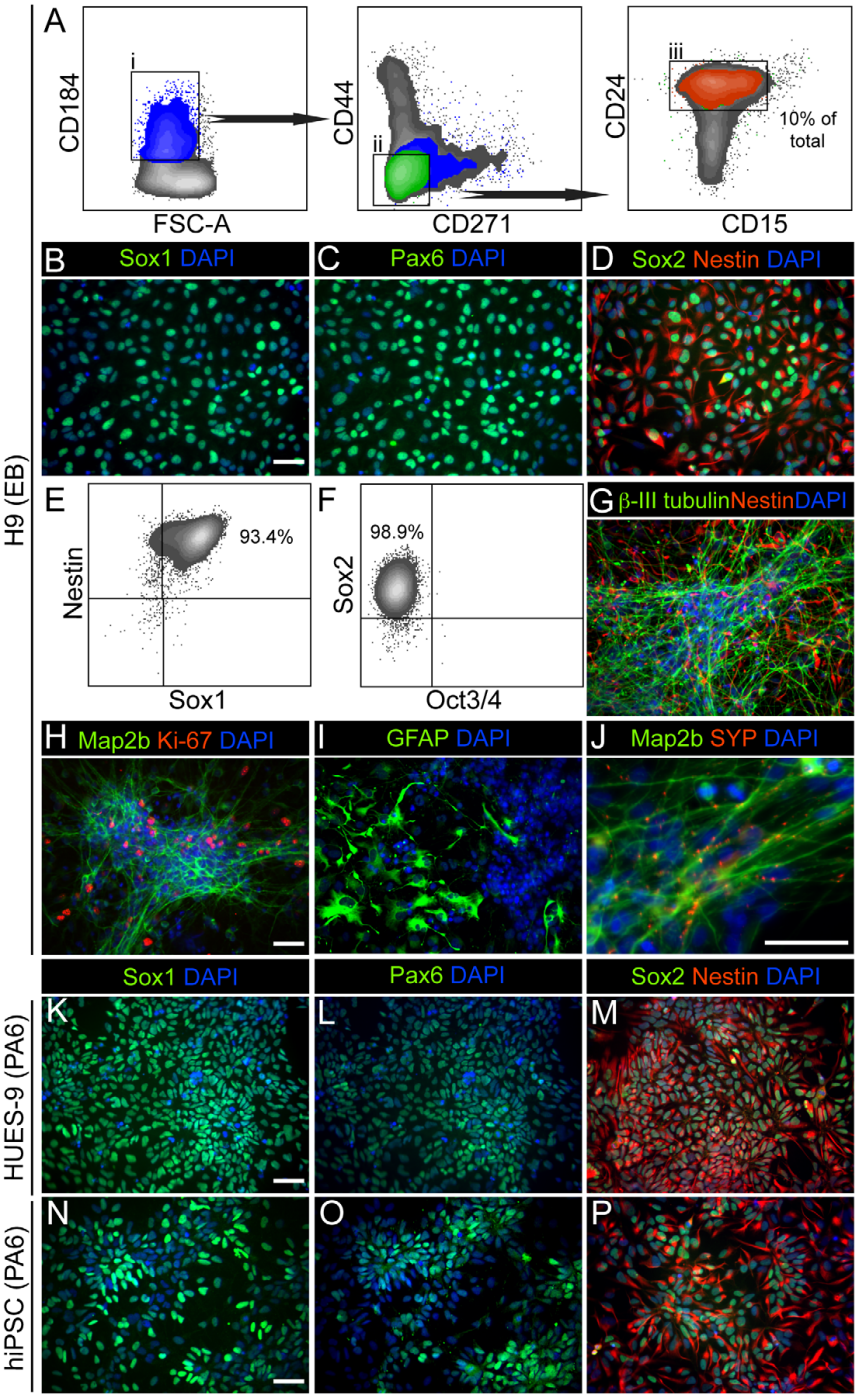
Sorting NSC from differentiating hESC and hiPSC from SFEB and SDIA neural induction cultures. (A) Cell sorting strategy of NSC derived from H9 SFEB cultures at the EB-rosette(+) stage. Cells were first selected based on CD184 staining. The CD184^+^ population was then depleted of cells expressing CD271 and CD44. This cell population was nearly 100% positive for CD24 and contained cells expressing CD15. 10% of the total cells (orange) were CD184^+^/CD271^−^/CD44^−^/CD24^+^. (B and C) Staining with anti-Sox1, anti-Pax6 and DAPI of CD184^+^/CD271^−^/CD44^−^/CD24^+^ H9 NSC from SFEB neural induction cultures at the third passage post-FACS. (D) Same as B but stained with anti-Sox2, anti-Nestin, and DAPI. (E and F) Four color intracellular FACS analysis with anti-Sox1, anti-Nestin, anti-Sox2 and anti-Oct3/4 of CD184^+^/CD271^−^/CD44^−^/CD24^+^ H9 NSC from SFEB at the third passage post-FACS. (G) CD184^+^/CD271^−^/CD44^−^/CD24^+^ H9 NSC expanded to passage 7 and induced to differentiate for 3 weeks and stained with anti-Nestin, anti-β-III tubulin and DAPI. (H-J) The CD184^+^/CD271^−^/CD44^−^/CD24^+^ H9 NSC were differentiated for 3 weeks and stained with anti-Map2b, anti-Ki-67 and DAPI; anti-GFAP, anti-synaptophysin (SYP) and DAPI. (K and L) Staining with anti-Sox1, anti-Pax6 and DAPI of CD184^+^/CD271^−^/CD44^−^/CD24^+^ HUES-9 NSC from SDIA PA6 neural induction cultures at the fourth passage post-FACS. (m) Same as k but stained with anti-Sox2, anti-Nestin and DAPI. (N-P) Same as K-M but with hiPSC, NDC3.1 NSC from SDIA PA6 neural induction cultures. Scale bar is 50 µm.

From our FACS screen we hypothesized that CD184^+^/CD271^−^/CD44^−^/CD24^+^ could be used for isolating a population of NSC from neural induction cultures while excluding hESC, NSC contaminants, EB-rosette(−) and CD271^+^ neural crest stem cells [34], [35] (Fig. 1B, C). We chose CD184 as a positive marker as it was not expressed in hESC but was expressed in NSC and identified a distinct subpopulation in EB-rosette(+). CD271 and CD44 were convenient negative markers as they were expressed on the majority of hESC, NSC contaminants and cells in the EB-rosette(−) culture. CD271 also provided the added advantage of removing neural crest. The cell sorting strategy used is depicted in Fig. 2A. Similar to “good” preparations of manually isolated NSC, these cultures were often free of NSC contaminants and could be cultured in vitro for multiple passages (up to 22 tested). Image and/or intracellular FACS analysis demonstrated that sorted cells were negative for Oct3/4 and were highly enriched for cells expressing Sox1, Sox2, Pax6 and Nestin (Fig. 2B–F). CD184^+^/CD271^−^/CD44^−^/CD24^+^ cells could be induced to differentiate to heterogeneous populations of neurons, glia and undifferentiated NSC. Image analysis revealed expression of neuronal markers β-III tubulin, Map2b and synaptophysin; the NSC marker, Nestin and the proliferation marker Ki-67 (Fig. 2G–J). Similar to manually isolated NSC, GFAP-immunoreactive astrocytes were only observed after 1 month of differentiation (Fig. 2I).

We tested whether the CD184^+^/CD271^−^/CD44^−^/CD24^+^ signature applied to other cell lines and neural induction methods. Using this signature we isolated NSC derived from SFEB neural induction cultures of a hiPSC line, NDC3.1. These cells exhibited similar qualities to those derived from H9. They expressed Sox1, Pax6, Sox2 and Nestin, and they were positive for Ki-67 (Fig. S3A–C). We isolated NSC from H9 SFEB cultures or monolayer differentiation cultures that were treated with recombinant noggin and the SMAD inhibitor SB431542 [4], [8]. The percentage of CD184^+^/CD271^−^/CD44^−^/CD24^+^ cells in SMAD-inhibited monolayer cultures was similar to SFEB cultures (10-15% of total), whereas combined dual SMAD inhibition with SFEB led to an increase in this NSC population (∼25% of total) (Fig. S3D,E and data not shown). We were also successful in isolating NSC derived from a hiPSC line, NDC3.1, and the hESC line HUES-9 from stromal derived induction activity (SDIA) neural induction cultures using the PA6 mouse stromal cell line [6], [7], [36]. These cells expressed Sox1, Pax6, Sox2 and Nestin (Fig. 2K–P). In some experiments, NSC isolated from SDIA cultures were resorted after the first passage to remove NSC contaminants that spontaneously appeared (data not shown). This problem was most common with HUES-9. NSC contaminants were usually not observed in resorted cultures 10 passages after resorting (data not shown).

We tested whether CD184^+^/CD271^−^/CD44^−^/CD24^+^ selected for all of the NSC in neural induction cultures by sorting the cells excluded by CD184^+^/CD271^−^/CD44^−^/CD24^+^, and analyzing them for expression of the neural stem cell markers Sox1, Sox2 and Pax6 by intracellular FACS (Fig. S3F). We observed a Sox1^+^/Sox2^+^/Pax6^+^ cell subpopulation in the excluded cells from H9 SFEB cultures. Similar trends were observed for HUES-9 and NDC3.1 SDIA neural induction cultures (data not shown). These data indicate that CD184^+^/CD271^−^/CD44^−^/CD24^+^ selects for a subpopulation of NSC rather than all of the NSC from heterogeneous neural induction cultures.

We tested whether CD184^+^/CD271^−^/CD44^−^/CD24^+^ NSC maintained expression of NSC markers and maintained neurogenic potential after multiple passages in culture (Fig. S4). Intracellular FACS analysis demonstrated that CD184^+^/CD271^−^/CD44^−^/CD24^+^ cells derived from H9, HUES-9 and NDC3.1 expressed high levels of Sox2 and Nestin at passages 19, 22 and 16, respectively (Fig. S4A–C). CD184^+^/CD271^−^/CD44^−^/CD24^+^ NSC derived from H9 maintained neurogenic potential up to 22 passages tested (Fig. S4D). We counted 100 DAPI-positive cells for 3 replicates of passage 22 cells differentiated for 3 weeks. We found that 42 (+/−10) percent of the cells were β-III tubulin positive. We also assessed neuronal differentiation in passage 19 NSC derived from H9 by intracellular FACS (Fig. S4E). Neurons could be identified by a Sox2^−^/Nestin^−/LOW^ population that expressed the neuronal marker doublecortin (DCX), but did not express Ki-67, and were found in similar numbers compared to image quantification. Image analysis of differentiated HUES-9 NSC revealed expression GFAP-positive astrocytes and neurons expressing the mature neuronal markers Map2a/b, synapsin and GABA (Fig. S4F-I). We quantified the percentage of differentiated HUES-9 NSC committed to the neuronal fate by counting β-III-tubulin positive cells. We counted 100 DAPI-positive cells from two different experiments with two biological replicates. 31 (+/−7) percent were β-III-tubulin positive. We also assessed the percentage of neurons in differentiated NDC3.1 NSC cultures by intracellular FACS and observed similar trends (Fig. S4J). Mature oligodendrocytes, based on both marker expression and morphology, were not detected in our in vitro NSC differentiation cultures (data not shown).

We performed a clonal assay to evaluate if a single NSC could give rise to a multipotent NSC population (Fig. S4K–N). CD184^+^/CD271^−^/CD44^−^/CD24^+^ NSC derived from NDC3.1 were sorted into 96 well plates at one cell per well on a feeder layer of mitotically inactivated mouse astrocytes to improve the survival of the single cells. We observed colonies of 20 cells or more appearing between 2-3 weeks after plating (data not shown). The subcloning efficiency was 3 percent. The clonal cell population expressed Sox2 and hNA (Fig. S4K). We differentiated the cells for 3 weeks after clones were visible to determine if they could give rise to neurons and glia (Fig. S4L–N). We observed co-localization of hNA with both GFAP and β-III tubulin, demonstrating that CD184^+^/CD271^−^/CD44^−^/CD24^+^ cells are multi-potent NSC.

### CD184 and CD44 define a cell surface marker signature for the purification of neurons and a glial cell population from differentiating NSC cultures

From the imaging screen of differentiated NSC and subsequent hit verification by FACS, we predicted that CD184^−^/CD44^−^/CD15^LOW^/CD24^+^ would identify a population of neurons, whereas CD184^+^/CD44^+^ would identify a population of glia. CD184 was expressed in the processes of some neurons but not in the cell bodies as previously reported in human fetal neural progenitor differentiation [37] (Fig. 1D,E). As processes are cleaved during the process of obtaining a single cell suspension, the CD184-positive signal is lost from the majority of the neurons. CD44 has been shown to be a marker of both glial progenitors and astrocytes in many species; therefore, we hypothesized that the CD44 subpopulation in our cultures contained these cell types [16], [38], [39]. CD15 and CD24 were added to the neuronal sorting strategy for confirmation and further refinement of the population.

**Figure 3:**
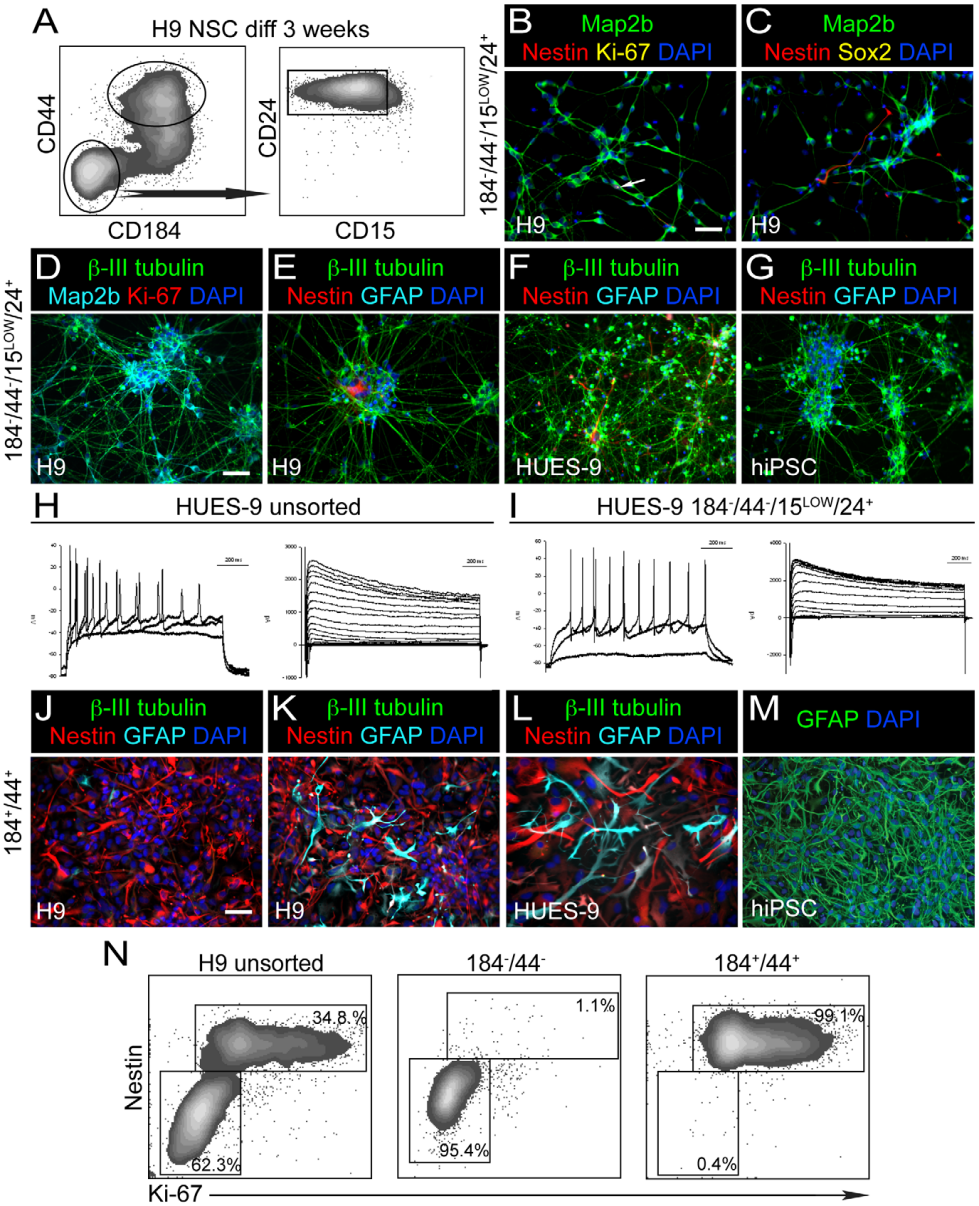
Sorting neurons and glia from cultures of sorted and subsequently expanded and differentiated NSC. NSC were differentiated for 3 weeks in neuron differentiation medium prior to FACS. (A) Cell sorting strategy of differentiated H9 NSC using CD184^−^/CD44^−^/CD15^LOW^/CD24^+^ and CD184^+^/CD44^+^. Similar populations were isolated from differentiated cultures of HUES-9 NSC and NDC3.1 NSC both derived from SDIA PA6 co-culture. (B) Sorted CD184^−^/CD44^−^/CD15^LOW^/CD24^+^ cells were cultured in neuron differentiation medium for 2 days post-FACS and subsequently stained with anti-Map2b, anti-Nestin, anti-Ki-67 and DAPI. White arrow indicates the presence of one Ki-67+, Nestin+ cell in this field. (C) Same as B except cells were stained with anti-Sox2 instead of Ki-67. One Nestin+ cell is evident in this field. (D) CD184^−^/CD44^−^/CD15^LOW^/CD24^+^ sorted cells were cultured in neuron differentiation medium for 7 days post FACS and subsequently stained with anti-β-III tubulin, anti-Map2b, anti-Ki-67 and DAPI. No Ki-67+ cells are observed in this field. (E) Same as D but stained with anti-β-III tubulin, anti-Nestin, anti-GFAP and DAPI. No GFAP+ cells are evident and one Nestin+ cell is evident in this field. (F) Same as E but from HUES-9. (G) Same as E but from hiPSC, NDC3.1. (H) Electrical recordings of patched neurons from a 3-week differentiated HUES-9 NSC culture. All cells exhibited Na^+^ current (right panel). 7 of 8 cells tested fired action potentials when depolarized with current (left panel). (I) Electrical recordings of sorted neurons from a 3-week differentiated HUES-9 NSC culture. Sorted neurons were cultured for an additional 3 weeks prior to recording. All cells exhibited Na^+^ current (right panel). 6 of 8 cells fired action potentials when depolarized with current (left panel). (J) CD184^+^/CD44^+^ sorted cells derived from H9 were cultured in neuron differentiation medium for 7 days post-FACS and stained with anti-β-III tubulin, anti-Nestin, anti-GFAP and DAPI. (K) Same as H except cells were cultured in astrocyte culture medium for 7 days prior to imaging. (L) Same as K, but from HUES-9. (M) CD184^+^/CD44^+^ cells from NDC3.1 were cultured for 6 passages in astrocytes medium and stained for GFAP and DAPI. Scale bar is 50 µm. (N) 2-color intra-cellular FACS analysis of NSC sorted from H9 and then induced to differentiate for 3 weeks prior to sorting cell populations based on different combinations of CD184 and CD44 immunoreactivity. The unsorted population is composed of a Nestin^+^ population that is highly enriched for mitotic Ki-67^+^ cells whereas the Nestin^−/LOW^ population is quiescent. Sorting of a CD184^−^/CD44^−^ population enriches for Nestin^−/LOW^ quiescent cells. Isolation of CD184^+^/CD44^+^ and CD184^+^/CD44^−^ cells enriches for the Nestin^+^ cycling population. Percentages for these 2 populations are indicated.

Figure 3A illustrates the cell sorting strategy we employed in our experiments. We isolated CD184^−^/CD44^−^/CD15^LOW^/CD24^+^ cells and CD184^+^/CD44^+^ cells simultaneously from cultures of H9 NSC (sorted from SFEB cultures) that were differentiated for 3 weeks. Two days post-FACS the CD184^−^/CD44^−^/CD15^LOW^/CD24^+^ cell population was largely composed of neurons that expressed Map2b and contained few cells expressing Nestin, Sox2 or Ki-67 (Fig. 3B,C). At 7 days post-FACS, the majority of the cells were β-III tubulin-, Map2b-positive polarized neurons with extensive arborized processes (Fig. 3D). This cell population was devoid of GFAP-expressing astrocytes, and few Nestin-positive or Ki-67-positive cells could be observed (Fig. 3D,E). Some sorted neurons expressed the neuronal markers tyrosine hydroxylase, GABA and synaptophysin, suggesting that mature neurons were present in these cultures (Fig. S5D-F). Similar results were obtained from sorting comparable differentiated NSC cultures derived from HUES-9 and NDC3.1, as described in Figure 2 and Figure S3 (Fig. 3F,G and data not shown).

To assess the purity of CD184^−^/CD44^−^/CD15^LOW^/CD24^+^ sorted neurons, we quantified expression of β-III tubulin, Nestin and Ki-67 7 days post-FACS by image analysis. We quantified 3 experiments from differentiated NSC derived from H9, HUES-9 and NDC3.1. We counted 3 random fields; 300 DAPI positive cells were counted for each experiment. The means for β-III tubulin, Nestin and Ki-67 for the 3 experiments were 98 (−/+ 1), 1.6 (−/+ 1) and 0.6 (−/+ 0.9) percent, respectively.

Similar to neurons in unsorted cultures, sorted neurons were electrophysiologically active. All of the cells tested had sodium and potassium currents, and 6/8 of the cells patched fired action potentials (Fig. 3H, I). Surprisingly, sorted neurons were modestly viable without supporting cells and could be cultured in vitro for at least 1 month with approximately 20–30% of the initial population remaining (Fig. S5A–C). Moreover, sorted neurons could be co-cultured with both human and mouse astrocyte cultures (Fig. S5G,H). Together, these data illustrate the utility of this sorting method for generating highly pure populations of functional neurons for downstream analysis under different culturing conditions.

The CD184^+^/CD44^+^ population was isolated from differentiated NSC cultures derived from H9, HUES-9 and NDC3.1 (Fig. 3J–M). At 7 days post FACS, this population appeared to be homogeneous for Nestin-immunoreactive cells, with few, if any β-III tubulin-positive neurons or GFAP-positive astrocytes (Fig. 3J and Fig. S5k). Many of these cells expressed Ki-67, demonstrating that they were mitotic (data not shown). At 14 days post-FACS, neurons were absent but many GFAP-positive astrocytes were observed (data not shown). These data suggested that the CD184^+^/CD44^+^ population was restricted in its differentiation capacity to glial lineages. When cultured in medium formulated for astrocyte culture, robust GFAP expression could be observed 7 days post-FACS, further demonstrating their gliogenic potential (Fig. 3K, L). We evaluated the fate of CD184^+^/CD44^+^ derived from NDC3.1 after 6 passages in culture in astrocyte media. Image analysis revealed that the majority of the cells expressed GFAP (Fig. 3M). We subsequently cultured these cells in neuron differentiation media for 2 weeks to determine if the cells had potential to differentiate to oligodendrocytes and neurons, but did not detect these cell types (data not shown). These data suggest that the CD184^+^/CD44^+^ cell population is enriched in cells that can mature to GFAP-expressing astrocytes.

Together, these data suggested that in differentiating cultures of NSC, CD184^−^/CD44^−^/CD15^LOW^/CD24^+^ identified a population of post-mitotic neurons and CD184^+^/CD44^+^ identified a population of glia. To further assess the purity of these populations we analyzed sorted cell populations by intracellular FACS for Nestin and Ki-67 expression immediately after cell sorting (day 0 post-FACS) (Fig. 3N). We eliminated CD15 and CD24 from this sort to allow for staining with these additional antibody conjugates. In unsorted 3-week differentiation cultures of NSC we observed Nestin^−/LOW^ and Nestin^+^ populations. The Nestin^−/LOW^ population was largely composed of quiescent cells, lacking Ki-67 expression [40], whereas the Nestin^+^ population was largely mitotic based on Ki-67 expression. Analysis of the sorted populations revealed that the majority of the CD184^−^/CD44^−^ cells were Nestin^−/LOW^/Ki-67^−^ post-mitotic neurons and that the CD184^+^/CD44^+^ cells were a near-homogeneous population of mitotic Nestin^+^ cells.

### Survival, maturation and phenotype of spinally grafted NSC populations

**Figure 4:**
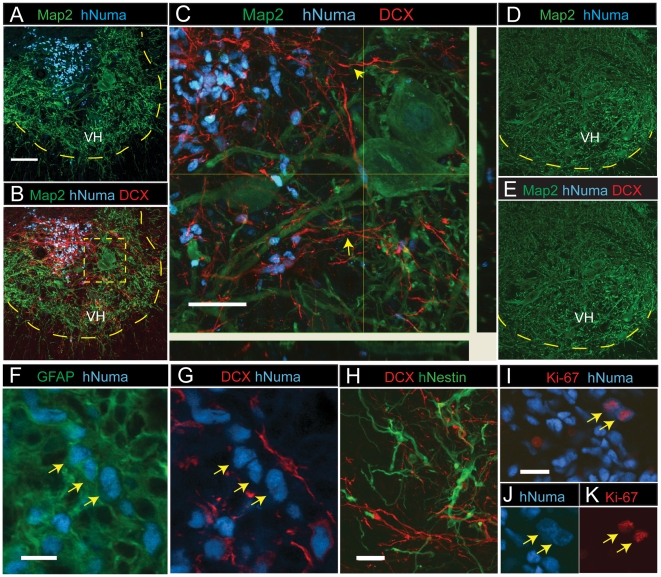
Spinal transplantation of differentiated NSC cultured cells in rats with spinal ischemic injury. (A) In animals with previous ischemic injury hNUMA+ grafted cells (blue) were identified in the intermediate zone or in the ventral horn (VH) 2 weeks after grafting. Scale bar is 100 µm. (B, C) Numerous hNUMA+ cells were DCX immunoreactive and showed extensive projection of DCX+ processes towards the ventral horn (yellow arrows). Scale bar is 40 µm. Yellow dotted box represents expanded view in C. (D, E) In control animals injected with medium only, no hNUMA or DCX immunoreactivity was identified. (F, G) A subpopulation of grafted hNUMA-positive cells showed colocalization with GFAP but were DCX-negative (yellow arrows). Scale bar is 10 µm. (H) At 2 weeks after grafting hNestin-positive cells were seen in the core of the graft and were DCX negative. Scale bar is 20 µm. (I–K) Proliferating cells were identified by colocalization of Ki-67 and hNUMA immunoreactivity and were primarily seen 2 weeks after grafting. Scale bar is 20 µm.

To assay functional engraftment abilities, HUES-9 SDIA cells purified by sorting with the CD184^+^/CD271^−^/CD44^−^/CD24^+^ signature were transferred to differentiation conditions for 3 weeks and the resulting cultures were transplanted as a single-cell suspension into spinal ischemia-injured rats. The presence and phenotype of grafted cells were analyzed 2 or 4 weeks after spinal grafting. Double staining with human-specific nuclear antibody (hNUMA) and anti-DCX, which stains immature post-mitotic neurons, revealed numerous terminally differentiated grafted neurons with extensive DCX-immunoreactive processes projecting towards the ventral horn as well as medially and laterally in the central gray matter (Fig. 4A–C; yellow arrows). No hNUMA or DCX immunoreactivity was seen in animals injected with medium only (Fig. 4D,E).

Confocal analysis of anti-hNUMA and anti-GFAP double-stained sections revealed that numerous grafted cells were also GFAP-immunoreactive and were typically localized at the periphery of individual grafts (Fig. 4F; yellow arrows) and were DCX-negative (Fig. 4G). Staining with anti-human specific Nestin (anti-hNestin) revealed a subpopulation of grafted cells that were Nestin-immunoreactive 2 weeks after grafting. These cells were typically localized in the center of grafts and were DCX-negative (Fig. 4H). At 4 weeks after grafting only occasional hNestin-immunoreactive cells were identified. Staining with an antibody for more mature human neurons, anti-human specific neuron-specific enolase, showed no immunoreactivity in grafted cells after 2 or 4 weeks. Similarly, only occasional human-specific synaptophysin staining was seen at this time point (data not shown), indicating that grafted neurons were immature at this stage, which is consistent with the observed intense DCX staining. To identify proliferating cells, sections were stained with anti-Ki-67 and co-stained with anti-hNUMA. Cells containing Ki-67 and hNUMA reactivity were identified both in the core of the graft and the periphery of the grafted area (Fig. 4I-K). Several Ki-67-positive, hNUMA-positive cells were seen 2 weeks after grafting but fewer were seen 4 weeks after grafting.

**Figure 5:**
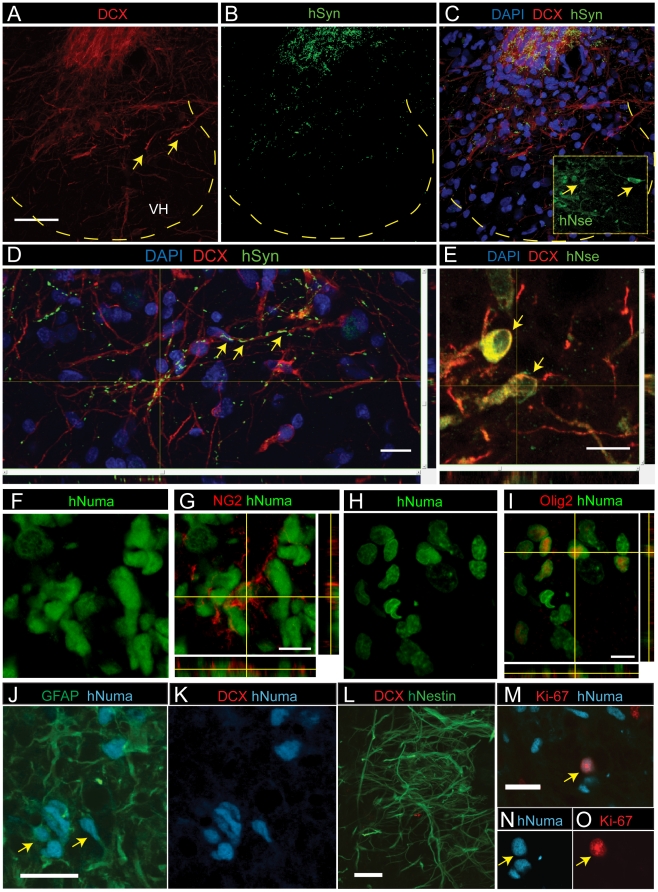
Spinal transplantation of proliferating NSC cultured cells in rats with spinal ischemic injury. (A) At 10 weeks after grafting a high density of DCX+ grafted neurons (red) in the center of intermediate zone were identified. Numerous solitary DCX+ neurons which migrated into ventral horns (yellow arrows) were also seen. Scale bar is 100 µm. (B, C) High density of hSYN punctata-like immunoreactivity in the regions containing grafted DCX+ neurons was seen. (D) hSYN immunoreactivity showed spatial co-localization with DCX+ processes (yellow arrows). Scale bar is 20 µm. (C-insert, E) Numerous hNSE immuoreactive neurons in the core of grafted regions were identified (yellow arrows). Scale bar is 20 µm. (F–I) A subpopulation of grafted cells acquired an oligodendrocyte phenotype and were NG2 or Olig2 immunoreactive. Scale bar is 20 µm. (J, K) Transgenic SOD1 mutant rats were grafted with CD184^+^/CD44^+^ sorted glial cells derived from differentiated HUES-9 NSC cultures. Numerous hNUMA/GFAP-positive astrocytes were identified in the core of the graft 2 weeks after grafting but no DCX-positive neurons were seen. Scale bar is 20 µm. (L) 2 weeks after grafting numerous hNestin-positive cells typically localized in the core of the graft. Scale bar is 20 µm. (M–O) Costaining with hNUMA and Ki-67 antibody revealed occasional proliferating cells (yellow arrow). Scale bar is 20 µm.

We next tested long-term engraftment and differentiation of CD184^+^/CD271^−^/CD44^−^/CD24^+^ -sorted proliferating NSC. Cells were expanded for 5-8 passages, grafted into lumbar spinal segments in immunosuppressed ischemia-injured rats and animals were sacrificed 8–10 weeks after grafting. In addition to DCX staining sections were also stained with markers expressed in mature neurons including human-specific enolase (hNSE) and human-specific synaptophysin (hSYN). Similarly as for induced NSC a high number of DCX immunoreactive grafted neurons were identified at 10 weeks after cell transplantation (Fig. 5A). In addition an intense hNSE and hSYN punctate-like immunoreactivity was seen in grafted neurons (Fig. 5B-D) suggesting substantial numbers of more mature neurons than seen at shorter times after transplant. Co-staining with DCX and hNSE antibody showed a subpopulation of grafted neurons co-expressing both markers (Fig. 5E). These data demonstrate that proliferating NSC maintain their neurogenic potential after in vivo grafting and show progressive expression of markers that are characteristics of mature neurons. Staining with NG2 and Olig 2 suggest that a subpopulation of grafted cells acquired an oligodendrocyte phenotype (Fig. 5F–I).

To test glial differentiation, we assessed the ability of CD184^+^/CD44^+^ cells to engraft in transgenic SOD1 mutant rats, where transplantation of glial progenitors has been shown to be neuroprotective [41]. Analysis of spinal cord sections 2 weeks after grafting and stained with anti-hNUMA and anti-GFAP showed numerous GFAP-positive grafted cells (Fig. 5J; yellow arrows). No DCX-immunoreactive cells were observed (Fig. 5K). Staining with anti-hNestin revealed the presence of numerous hNestin-positive cells in the core of the graft at 2 weeks after cell grafting (Fig. 5L). Costaining with anti-hNUMA and anti-Ki-67 revealed the presence of some proliferating grafted cells (Fig. 5M–O). These data demonstrate that CD184^+^/CD44^+^ cells can differentiate to astrocytes *in vivo*.

We also tested the ability of CD184^−^/CD44^−^/CD15^LOW^/CD24^+^ neurons to engraft in spinal ischemia-injured rats (data not shown). In 2 separate experiments we observed poor engraftment by evidence of inflammation and dead cells. These results are consistent with previous findings that show superior survival of grafted fetal brain tissue or embryonic tissue-derived neural or neuronal precursor cell lines [42], [43], [44], [45].

Finally, while additional extensive and long-term survival studies need to be performed to assess precisely the tumor-forming potential of the cells we purify, our initial results demonstrate that no tumor-like structures or clusters of aberrant-proliferating cells were identified in any animal grafted with proliferating or differentiated NSC cultures or glia. To date we have analyzed more than 40 transplanted immunosuppressed Sprague-Dawley rats or immunodeficient rats and 6 immunosuppressed minipigs surviving for up to 3 months after cell grafting. No tumor formation was observed in any of these animals. These data indicate that CD184^+^/CD271^−^/CD44^−^/CD24^+^ NSC isolated from pluripotent stem cells represent a source of NSC that may be safer and thus useful in cell replacement strategies.

## Discussion

**Figure 6:**
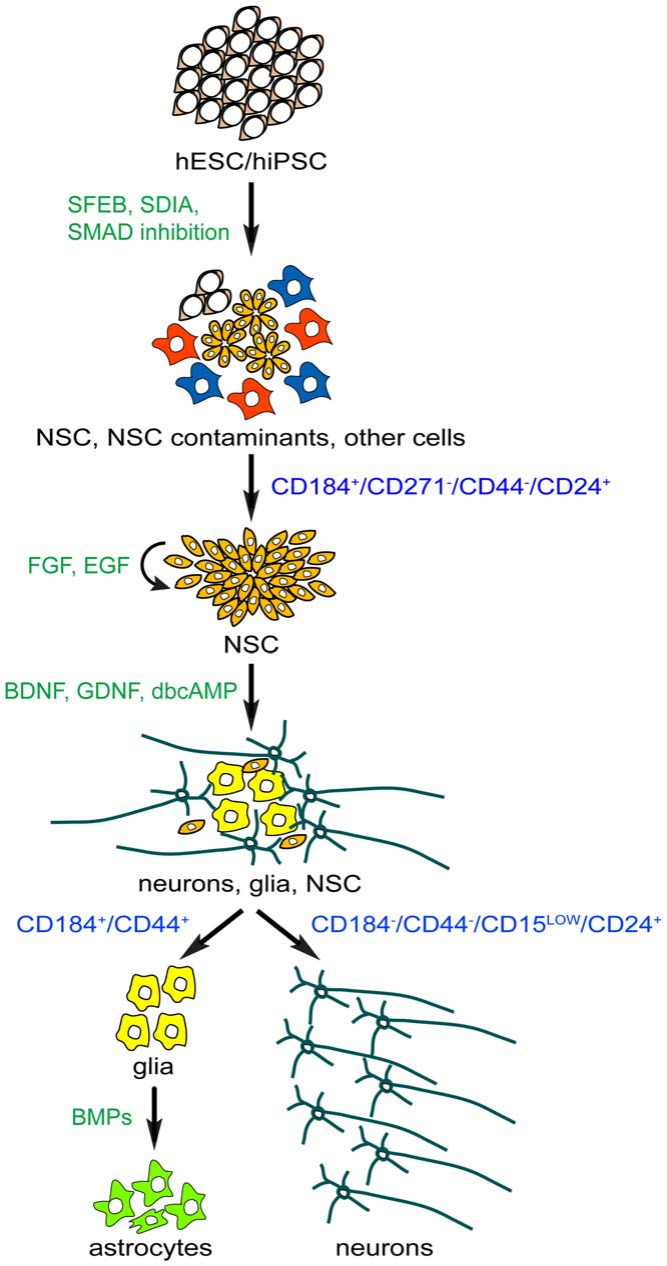
Diagram of stages and defined markers for isolation of NSC, neurons and glia from neural induction cultures starting with pluripotent stem cells.

We discovered a set of cell surface signatures that enable the isolation of NSC, neurons and glia derived from neural induction cultures of pluripotent stem cells. Figure 6 illustrates a schematic of our cell sorting methods. An important advantage of our approach is that it enables more consistent NSC preparations by allowing for the isolation of NSC without manual dissection of rosettes. In addition, the sorted glial cells provide a population of replicating astrocytes that can be subsequently cultured in vitro. Most importantly, our approach allows for the isolation of near-pure populations of terminally differentiated neurons that are viable in culture. In addition to cell sorting, these cell surface signatures can also be used to comparatively analyze cell populations. In addition, to the hits we further analyzed, there are many additional markers from our data set to explore that could potentially identify unique subpopulations of NSC, neurons and glia.

Our unbiased screen suggests novel uses of known markers for the isolation of neural cell types. CD184^+^ has been described for the isolation of neural cells capable of differentiating to neurons post-FACS [27]. We observed that CD184 negative selection, not positive selection was required for isolation of post-mitotic neurons. Another study reported the enrichment of hESC-derived neurons by isolating CD56^+^ neurons by FACS, but purity was not described [26]. Based on our screening data, CD56^+^ would not adequately discriminate against non-neuronal cells. Pruszak et al. (2009) performed a cell surface marker analysis on expanded and differentiated neural rosettes and reported that CD29, CD15 and CD24 defined distinct cell types within this cell population [30]. They described multiple populations based on CD24 immunoreactivity, which we did not observe, possibly due to the differences in the maturation of the neural induction cultures used. It will be interesting to determine the relationship of their signatures to those described here. CD133 has proven useful for isolating NSC in multiple studies [28]. We avoided CD133 as a selection marker for NSC due to its high expression in hESC and dim expression in EB-rosette(+) cultures in our screens, which raises the risk of insufficient purification away from pluripotent stem cells. In addition to neural ectoderm, CD184 and CD15 have been shown to mark and isolate progenitors from other germ layers [46], [47], [48], [49]. Since we could not examine all possible differentiation methods, more work is needed to determine whether the signatures described here are sufficient for isolating neural cell types in additional in vitro differentiating cell culture systems. Additional negative selection markers may be necessary for discriminating other cell types from very heterogeneous cell cultures. For example, Flk-1 may be useful for excluding mesodermal cells [46]. Comparison of our data set to those of subsequent immunophenotyping screens on endodermal and mesodermal cell lineages may ultimately lead to appropriate cell-surface signatures for desired cell types in any culture condition.

We believe that immunophenotyping screens could be performed in higher throughput and greater complexity. For example, it may be possible to take advantage of advances in fluorescent cell bar-coding to enable immunophenotyping of multiple cell cultures simultaneously in one experiment [50]. We were successful in correlating neuronal β-III tubulin staining with cell surface markers by image analysis. It may be possible to combine cell-surface immunophenotyping with intracellular FACS analysis to identify cell-surface signatures of specific cell types and subpopulations therein. This type of analysis could be instrumental in defining cell surface marker signatures for different developmental stages in neural induction and regional specification, as well as for specific cell subtypes such as dopaminergic neural progenitors and neurons.

Our grafting studies of 3-week differentiation cultures of CD184^+^/CD271^−^/CD44^−^/CD24^+^ HUES-9 NSC revealed a time course of neuronal and glial maturation and a clear population of grafted DCX-positive neurons or GFAP-positive astrocytes identified 2-4 weeks after grafting, consistent with our previous studies [45], [51], [52]. No hNSE or human specific synaptophysin (hSYN) expression was seen at 4 weeks. In contrast, intense hNSE and hSYN immunoreactivity was seen 8–10 weeks after grafting of proliferating NSC, similar to the neuronal maturation profile of proliferating human fetal spinal neural precursors grafted spinally in the same spinal-ischemia model or in naïve immunosuppressed minipig in earlier studies [45], [53]. In addition, a subpopulation of grafted proliferating NSC acquired oligodendrocyte phenotype as evidenced by expression of NG2 and Olig2. In contrast to long-term survival and maturation of grafted differentiated or proliferating NSC, no survival of CD184^−^/CD44^−^/CD15^LOW^/CD24^+^ purified neurons was seen at 2 or 4 weeks after grafting. These data suggest that transplantation of proliferating CD184^+^/CD271^−^/CD44^−^/CD24^+^ NPC is sufficient for long-term neuronal engraftment without tumor formation.

In transgenic SOD1 mutant animals grafted with CD184^+^/CD44^+^ glia, no DCX-immunoreactive neurons were identified and the majority of grafted cells expressed GFAP, similar to our long term in vitro culture. More extensive studies need to be performed to determine whether CD184^+^/CD44^+^ sorted glia have potential to differentiate to oligodendrocytes, but at this time they appear to be highly enriched for cells that can give rise to astrocytes.

While we observed cells that expressed oligodendrocyte markers 10 weeks after grafting, we did not observe convincing staining with these markers our in vitro cultures. This difference could be niche and/or time dependent. Others have reported spurious oligodendrocyte differentiation of hESC-derived NSC in vitro [11]. We suspect that significantly longer culture times and/or different differentiation conditions may be required to achieve mature oligodendrocyte differentiation in vitro [54]. It is also possible that the CD184^+^/CD271^−^/CD44^−^/CD24^+^ NSC subpopulation is more biased to neuronal and astrocyte differentiation.

Our approach to defining cell surface signatures in differentiating cultures of pluripotent human stem cells will potentially enable quantitative benchmarks to be devised for comparing specific cell populations generated by different hESC and hiPSC lines. In addition, further antibody screens may yield cell surface signatures of specific neuronal and neural progenitor subtypes as well as signatures of desired cell types from other germ layers. For applications such as transplantation, our approach will be very useful for obtaining pure, well-defined cell populations. Finally, these methods are applicable to many studies of the characteristics of defined neuronal populations, expression analyses, generating defined mixtures of cell types to test disease models for non-cell-autonomous phenotypes, and for drug testing and development.

## Methods

### Ethics Statement

This study was approved by the University of California, San Diego (UCSD) Internal Review Board (IRB), approval ID# 100887. Written consents were obtained prior to biopsy for all research uses.

### hESC culture

For feeder growth conditions, H9 (WiCell) hESCs were cultured and maintained according to the methods recommended by WiCell. Briefly cells were cultured on a irradiated mouse embryonic fibroblast (MEF) feeder layer in DMEM:F12, 20% Knockout Serum Replacement (KOSR), non-essential amino acids, 20 mM glutamine (all from Invitrogen), penicillin/streptomycin (Lonza), bFGF (BD). Cells were passaged using collagenase IV (Invitrogen). For feeder-free growth conditions, H9 cells were cultured in mTeSR1 maintenance medium (Stem Cell Technologies) on Matrigel-coated plates and passaged with Dispase (BD). HUES-9 were cultured according to the method described in Cowan et al [2]. Briefly, HUES-9 were maintained on a MEF feeder layer in HUES hESC medium containing Knockout-DMEM (Life Technologies), 10% plasmanate, 10% KOSR, non-essential amino acids, 20 mM glutamax, penicillin/streptomycin (all from Invitrogen), 10 ng/ml bFGF (R&D Systems). Cells were passaged with Trypsin.

### Generation and culture of hiPSC

Adult human skin biopsies were obtained from volunteers at the Alzheimer's Disease Research Center, University of California, San Diego. Adult human dermal fibroblasts were transduced with retroviruses encoding Oct3/4, Klf4, Sox2, c-Myc overnight. On day 3, the transduced fibroblasts were treated with 20 mM VPA (Sigma) for 7 days. On day 4, the fibroblasts were plated onto a MEF feeder layer with HUES hESC medium. The colonies were picked at 2 weeks when visible. They were initially passaged by mechanical dissociation and subsequently adapted to Trypsin (Invitrogen). HiPSC lines were characterized for their ability to differentiate into cells of the 3 germ layers and for the expression of biomarkers for pluripotency.

### Neural induction by SFEB

Neural induction was performed essentially as previously described [10]. Briefly, hESC were treated with dispase 1 mg/ml for 10 minutes. The colonies were lifted off and washed with fresh WiCell hESC medium without FGF. Cells were transferred to low adherence culture dishes in WiCell hESC medium without FGF. After 4 days in culture, EBs were plated on matrigel-coated (BD Biosciences) plates in ITS medium: DMEM:F12 (Life Technologies), ITS supplement (Sigma) and penicillin/streptomycin (Lonza) for about 16 days. At this point, columnar rosette structures were either manually isolated and passaged every other day for 5–7 days to remove contaminating cells or NSC were sorted from this heterogeneous cell population. In some experiments, 500 ng/ml recombinant Noggin (R&D Systems) and 10 µM SB431542 (Tocris) were added at the onset of the experiment and continued through day 10 in ITS medium.

### Generation of EB that are depleted of neural ectoderm (EB-rosette(-)) and NSC contaminant

HESC H9 colonies were scraped from their substrate and cell clumps were triturated to small pieces with a pipette. Cells were cultured in low-adherence plates in DMEM:F12, 20% FBS for 1 week and plated on matrigel-coated plates in the same medium for an additional 2 weeks. Under these conditions few rosettes were observed, with many EB demonstrating islands of loose endothelial-like cells and large vacuoles. NSC contaminants were generated from a failed attempt to manually isolate NSC from SFEB.

### Cell surface antibody screen

Antibodies to human cell surface markers were lyophilized in 96-well plates at 0.5 µg/well with the exception of CD133 (similar plates are commercially available from BD Biosciences; see Fig. S2 for antibodies used and clone names. For screening by flow cytometry, cells were dissociated with Accutase (Innovative Cell Technologies) and resuspended in BD Pharmingen Stain Buffer (BD Biosciences) with the addition of 5 mM EDTA. Cells were dispensed into 96-well round bottom plates (BD Biosciences) at 160,000 to 500,000 cells per well. Antibodies were reconstituted with 1X PBS and cells were stained live on ice for 20 minutes. Cells were washed with Stain Buffer and then stained with APC goat-anti-mouse IgG secondary antibody (BD Biosciences) for 20 minutes. Cells were washed and analyzed on a LSRII HTS system (BD Biosciences). Data were analyzed with FlowJo software and Microsoft Excel 2007 for generation of heat maps. For immunophenotyping by bioimaging, cells were cultured on 96-well bioimaging plates (BD Biosciences). Antibodies were reconstituted in NSC differentiation medium and cells were stained live on ice for 20 minutes. Cells were washed and then stained with an Alexa Fluor® 647 goat-anti-mouse IgG secondary antibody (Molecular Probes) for 20 minutes. Cells were washed, fixed with 4% paraformaldehyde, washed and imaged on a BD Pathway 435 confocal bioimager.

### Neural induction by dual SMAD inhibition on monolayer cultures

H9 hESC were grown in mTeSR1 medium on matrigel-coated plates and passaged as single cells with Accutase (Innovative Cell Technologies). When cultures became 100% confluent, medium was changed to ITS medium with the addition of 500 ng/ml Noggin and 10 µM SB431542 for 7 days. Cells were collected by gently scraping monolayers. Cell clumps were collected and replated on matrigel-coated plates in ITS medium. At day 12 medium was replaced with NSC growth medium 2 (see below) for 3 days before sorting.

### PA6 differentiation/SDIA

PA6 cells were plated in a 10 cm dish at 1∶5 from a confluent dish. The next day, HUES-9 were plated onto the PA6 cells at 100,000 cells/ml in PA6 differentiation medium: Glasgow DMEM, 10% KOSR,1 mM Sodium Pyruvate, 0.1 mM Nonessential Amino Acids and 0.1 mM β-Mercaptoethanol (all from Invitrogen). Medium was changed every 3–4 days, then daily after day 10 for an additional 10 days. To enhance neural induction, some cultures were treated with 500 ng/ml Noggin (R&D Systems) and 10 µM SB431542 (Tocris) for 6 days starting on the day of plating cells.

### NSC culture

Manually isolated or sorted NSC were cultured on 20 µg/ml poly-L-ornithine and 5 µg/ml laminin (both from Sigma) (POL) coated plates. Two different NSC culturing media were used. HUES-9 and hiPSC NSC were cultured in NSC growth medium 1: DMEM:F12+Glutamax, 0.5X N2, 0.5X B27, 1X P/S, 20 ng/ml FGF. H9 NSC were cultured in NSC growth medium 2: DMEM:F12+Glutamax, 1X N2, 1 µl/ml B27 (both from Life Technologies), 20 µg/ml insulin, 1.6 g/l glucose (both from Sigma), penicillin/streptomycin (Lonza), 20 ng/ml bFGF, 20 ng/ml EGF (both from BD Biosciences) [11]. In both cases, media were changed every 2–3 days and cells were split with Accutase (Innovative Cell Technologies).

### NSC differentiation

NSC were seeded on POL plates in NSC growth medium for HUES-9 and hiPSC and NSC growth medium 2 for H9. When the dish was 70% confluent, medium was switched to neuron differentiation medium: DMEM:F12+Glutamax, 0.5X B27, 0.5X N2 (Invitrogen), 1X P/S (Lonza), 20 ng/ml BDNF, 20 ng/ml GDNF (both from Peprotech) and 0.5 mM dibutyryl cyclic AMP (Sigma) for 3 to 4 weeks. Medium was changed every 2-3 days.

### Immunofluorescent imaging of cells in culture

Cells were fixed with 4% paraformaldehyde. Cells were either permeabilized in 0.1% Triton or PermWash buffer (BD Biosciences). See Table S1 for a list of primary purified and fluorochrome-conjugates antibodies used and their dilutions. Primary unconjugated antibodies were stained with secondary antibodies goat anti-rabbit and goat anti-mouse conjugated to Alexa 488, 647, 555; Molecular Probes). Cell nuclei were counterstained with DAPI. Cells were imaged on a BD Pathway 435 confocal bioimager and analyzed with Attovision software (BD Biosciences).

### FACS – isolation of NSC from differentiating hESC and hiPSC cultures

Cells were dissociated with Accutase (Innovative Cell Technologies) for 20-40 minutes. The cells were broken up by triturating. Cells were washed once with PBS to remove the Accutase and treated in NSC growth medium with 100 units/ml DNase (Sigma) for 10 minutes at room temperature and strained through a 70 µm cell strainer (BD Biosciences). Cells were washed and resuspended in NSC sorting medium: NSC growth medium 1 or 2 with the addition of 0.5% BSA and 5 mM EDTA. Cells were stained with fluorochrome-conjugated antibodies for 20–30 minutes (see Table S1 for antibodies and their dilutions). Cells were washed and resuspended in NSC sorting medium at a concentration of 5 million cells/ml. Cells were sorted with a FACSAria II (BD Biosciences) with a 100 µm nozzle at approximately 20 PSI and cells were collected in NSC growth medium 1 or 2. Cells were centrifuged and resuspended in fresh growth medium for plating.

### FACS – isolation of neurons and glia from differentiating NSC cultures

NSC were induced to differentiate in NSC differentiation medium for 3–4 weeks. Cells were prepared the same as the NSC except that NSC differentiation medium was used in place of NSC growth medium. Sorted neurons and glia were plated at 100,000–200,000 cells per well in 96-well imaging plates (BD Biosciences) coated with 20 µg/ml poly-l-ornithine and 0.25 mg/ml matrigel. We found it crucial for sequential coating of these 2 substrates. Sorted glia were cultured in NSC differentiation medium or Astrocyte culture medium (Lonza), which expedited GFAP expression.

### Intracellular FACS analysis

Cells were dissociated with Accutase, washed with 1X PBS, fixed with 4% paraformaldehyde and permeabilized with Perm/Wash Buffer or Perm buffer III (both from BD Biosciences). Cells were stained with flurochrome-conjugated antibodies for 20–30 minutes and washed with Perm/Wash Buffer or Stain buffer (if permeabilized with Perm buffer III (BD Biosciences) (see Table S1 for antibodies and their dilutions). Background staining for antibodies was determined in negative cell lines and with matched flurochrome-conjugated isotype controls. Cells were analyzed on an LSRII flow cytometry system (BD Biosciences).

### Neuron viability Assay

Neurons were sorted and cultured as described above. Cells were counted prior to plating. Four to 6 hours after plating cells were harvested using Accutase and counted with a hemocytometer using Trypan blue extrusion (Sigma) or a Vi-CELL analyzer (Beckman Coulter). Percent recovery was determined by the cell numbers recovered divided by the number of cells initially plated.

### Clonal Analysis

CD184^+^/CD271^−^/CD44^−^/CD24^+^ NSC were dissociated with Accutase to single cells and sorted one cell per well by FACS ARIAII into a 96 well plate containing mitotically inactivated mouse astrocytes. Floxuridine was used to inactivate the mouse astrocytes. NSC growth medium with FGF was replenished every other day.

### Electrophysiology

Whole-cell perforated patch recordings were performed from sorted HUES-9 NSC that were differentiated in neuron differentiation medium for 3 weeks. The recording micropipettes (tip resistance 4–8 MΩ) were tip filled with internal solution (115 mM K-gluconate, 4 mM NaCl, 1.5 mM MgCl_2_, 20 mM HEPES, and 0.5 mM EGTA [pH 7.3]) and then back filled with internal solution containing amphotericin B (200 g/ml). Recordings were made using an Axopatch 200B amplifier (Axon Instruments). Signals were filtered at 2 kHz and sampled at 10 kHz. The whole-cell capacitance was fully compensated, whereas the series resistance was uncompensated but monitored during the experiment by the amplitude of the capacitive current in response to a 5 mV pulse. The bath was constantly perfused with fresh HEPES-buffered saline (115 mM NaCl, 2 mM KCl, 10 mM HEPES, 3 mM CaCl_2_, 10 mM glucose, and 1.5 mM MgCl_2_ [pH 7.4]). For current-clamp recordings, cells were clamped at a range of −60 to −80 mV. For voltage-clamp recordings, cells were clamped at −70 mV. All recordings were performed at room temperature.

### Induction of spinal ischemic injury

Transient spinal cord ischemia (10 min) was induced as previously described [55]. Briefly, a 2F Fogarty catheter (Am. V. Muller) was passed through the left femoral artery to the descending thoracic aorta to the level of the left subclavian artery in isoflurane (1.5–2%)-anesthetized SD rats. Distal arterial pressure (i.e., below the level of aortic occlusion) was monitored by cannulation of the tail artery (PE-50). Spinal cord ischemia was induced by inflation of the intra-aortic balloon catheter (0.05 ml of saline) and concurrent systemic hypotension (40 mm Hg) induced by blood withdrawal (10.5–11 cc into a heated (37°C) external reservoir) via a 20-gauge polytetrafluoroethylene catheter in the left carotid artery. The efficacy of the occlusion was demonstrated by an immediate and sustained drop in distal blood pressure. After 10-min ischemia, the balloon was deflated, and the blood was reinfused. Once the arterial blood pressure was stabilized (20–30 min after reflow), the arterial lines were removed and wounds closed. After ischemia, the recovery of motor function was assessed in 2-day intervals using a 21-point open field locomotor scale [56]. Only animals with a score of 0-4 (i.e., corresponding with chronic paraplegia) at 1–2 months after ischemia were used in the transplantation study.

### Preparation of differentiated NSC for spinal implantation

NSC cultures were induced to differentiate for 3 weeks in NSC differentiation media. Cells were treated with Accutase (Innovative Cell Technologies) for 20–30 min and a single cell suspension was prepared by a repeated (10–15 times) trituration using a 10 mL plastic pipette. The cell suspension was treated with a DNase solution for 10 min. To remove cell debris, the cell suspension was placed on an ovomucoid gradient (Worthington Biochemical Corp) and centrifuged at 70× g for 6 min. Cells were resuspended in PBS and a viability test was performed using Trypan blue extrusion test (Sigma). On average, a 75–90% viability rate was recorded. The cell concentration was adjusted to a final concentration of 7,000–10,000 viable cells in 0.5 µl. the cells were stored at 4°C and used for implantation without further manipulation. Just before spinal injections, the cell suspension was aspirated into a glass capillary using a 50 µl Hamilton syringe.

### Preparation of proliferating NSC for spinal implantation

Proliferating NSC cultures at 10–15 passages were treated with Accutase (Innovative Cell Technologies) for 20–30 min and a single cell suspension was prepared by a repeated (10–15 times) trituration using a 10 mL plastic pipette. The pre-grafting cell preparation was identical to that described for induced NSC.

### Preparation of hESC-NSC-derived neurons for spinal implantation

HUES-9 NSC cultures were induced to differentiate for 3 weeks in neuron differentiation medium. CD184^−^/CD44^−^/CD15^LOW^/CD24^+^ neurons were then sorted and cultured in presence of cAMP, GDNF and BDNF for 1–2 weeks and then prepared for grafting. The pre-grafting cell preparation was identical to that described for induced NSC.

### Preparation of hESC-derived glia for spinal implantation in SOD+ rats

HUES-9 NSC cultures were induced to differentiate for 3 weeks in neuron differentiation medium. Sorted CD184^+^/CD44^+^ cells were cultured in NSC growth medium for 3 days before grafting. The pre-grafting cell preparation was identical to that described for differentiated NSC.

### Spinal cord implantation of proliferating, differentiated and sorted NSC populations

Animals were anesthetized with 1.5–2% isoflurane (in room air), placed into a spinal unit apparatus (Stoelting) and a partial Th12–L1 laminectomy was performed using a dental drill (exposing the dorsal surface of L2-L5 segments). Using a glass capillary (tip diameter 80–100 µm) connected to a pressure-controlled microinjector (Stoelting), rats were injected with 0.5 µl of the differentiated NSC in PBS. The duration of each injection was 60 s followed by 30 s pause before capillary withdrawal. The center of the injection was targeted into the central gray matter (laminae V–VII) (distance from the dorsal surface of the spinal cord at L3 level: 1 mm) [52]. The rostrocaudal distance between individual injections ranged between 300–500 µm. Animals (n = 4–7 for each cell population) received a total of 10 bilateral injections (10 injections on the left and 10 injections on the right side). After implantation, the incision was cleaned with 3% H_2_O_2_ and penicillin/streptomycin mixture and closed in 2 layers.

### Immunosuppression

All animals were immunosuppressed with Prograf (FK506; Astellas Pharma) in combination with Cellcept (mycophenolate mofetil; Roche Pharmaceuticals). Treatment with both drugs started one day before cell grafting. Prograf was injected at dose of 3 mg/kg/day i.p. for the first 14 days and the dose was lowered to 1 mg/kg/day and continued for the duration of study. Treatment with Cellcept was initiated at a dose of 50 mg/kg/day i.p. for the first 2 days and then the dose was decreased to 30 mg/kg/day. Treatment with Cellcept was stopped at 7 days post-grafting.

### Perfusion fixation, tissue processing and immunohistochemistry

At the end of the survival periods, rats were terminally anesthetized with pentobarbital (100 mg/kg; i.p.) and transcardially perfused with heparinized saline (100 ml) followed by 4% paraformaldehyde in 0.1 M phosphate buffer (PB; 500 ml). The spinal cords were dissected and postfixed in the same fixative overnight at 4°C. After postfixation, tissue was cryoprotected in a graded sucrose solution (10, 20 and 30%). Frozen coronal spinal cord sections (20–30 µm) were then cut.

Free-floating sections were incubated at 4°C with primary antibodies (see Table S1 for antibodies and their dilutions). After incubation with primary antibodies, sections were washed 3 times in PBS and incubated with fluorescent-conjugated species-specific secondary antibodies raised in donkey (Alexa 488, 546, 647 (Invitrogen, Carlsbad, CA). For multiple labeling experiments, primary antibodies from different species were applied simultaneously, followed by application of secondary antibodies conjugated to different fluorescent markers. For general nuclear staining, DAPI was added to the final secondary antibody solution. After staining, sections were mounted on slides, dried at room temperature and covered with Prolong anti-fade kit (Invitrogen, Carlsbad, CA). Slides were analyzed with a Leica (DMLB) fluorescence microscope and a Zeiss AxioCam MR camera. Some images were captured with an Olympus Fluoview 1000 confocal microscope. All images were processed by Adobe CS3 (Adobe Systems).

## Supporting Information

Figure S1 **Characterization of cells generated from SFEB culture of H9.** (A) Rosette at the EB-rosette(+) stage stained with anti-Sox1 and DAPI. (B) Picked NSC expanded from EB-rosette(+) were stained with anti-Sox1 and DAPI. (C) Same as c but stained with anti-Sox2, anti-Nestin, and DAPI. (D, E) Four-color intracellular FACS analysis with anti-Sox1, anti-Nestin, anti-Sox2 and anti-Oct3/4 of NSC expanded from EB-rosette(+). (F) NSC induced to differentiate for 3 weeks and stained with anti-Nestin, anti-β-III tubulin and DAPI. (G-L) Bright field images of (G) high quality NSC, (H) NSC that are crowded by contaminants (white arrows = NSC, black arrow = contaminants), (I) contaminants at low density, (J) contaminants at high density, (K) EB-rosette(-) depleted of NSC that have been plated and allowed to expand and (L) EB-rosette(+) illustrating columnar rosette structures. Scale bar is 50µm for A-F, 100 µm for G-L.
(TIF)

Figure S2 **Heat map depicting the results from the FACS and image screens as well as subsequent verification of imaging hits by FACS.** The data are organized as percent positive (% +ve) for FACS. Antibody specificity and clone names separate the FACS and image data. EB-rosette(+) = EB with rosettes; EB-rosette(-) = EB depleted of rosettes; NSC = neuronal stem cell expanded from manually isolated EB-rosette(+). NSC contaminant = culture of intermittent contaminant of handpicked and expanded NSC cultures; Neurons and glia = NSC that have been differentiated for 3 weeks and are composed of mixed cultures of neurons, glia and undifferentiated NSC. Images were classified as negative or too dim to determine (Neg/dim) or apparent expression in neurons (pink), non-neuronal cells (Other, blue), or both neurons and non-neuronal cells (Both, purple). Selected hits from the imaging screen were analyzed by FACS.
(TIF)

Figure S3 **FACS and image data of sorted cells from different neural induction methods.** (A, B) Staining with anti-Sox1, anti-Pax6 and DAPI of CD184^+^/CD271^−^/CD44^−^/CD24^+^ NDC3.1 NSC from PA6 co-culture at the 4th passage after the sort. (C) Same as (a and b) but stained with anti-Ki-67, anti-Nestin, and DAPI. Scale bar is 50 µm. (D) Sorting of H9 after SMAD inhibition with SFEB method. Note that the percentage of likely NSC increases from 10 to 23%. Also CD44 + contaminants are reduced. (E) Sorting of H9 after SMAD inhibition of cells as a monolayer. (F) H9 SFEB cultures that were also treated with dual SMAD inhibition were stained for CD184^+^/CD271^−^/CD44^−^/CD24^+^ and the cells not selected by the signature were sorted and analyzed for Sox1, Sox2 and Pax6 by intracellular FACS. The two dimensional plot indicates the presence of Sox2^+^/Sox1^+^ cells (blue, 24.1% of total). The histogram demonstrates that the Sox2^+^/Sox1^+^ cells are also positive for Pax6.
(TIF)

Figure S4 **Characterization of long-term NSC cultures.** (A-C) Intracellular FACS analysis of NSC cultures: (A) hiPSC NDC3.1 NSC at passage 16, (B) H9 at passage 19 and (C) HUES-9 at passage 22. For these studies we used a new Sox2 antibody that revealed two distinct Sox2 populations. (D) Immunofluorescent image of H9 sorted NSC passage 22 differentiated for 3 weeks and stained with β-III tubulin and DAPI. (E) Intracellular FACS analysis of H9 sorted NSC passage 19 differentiated for 4 weeks with Nestin, Sox2, DCX and Ki-67. (F) Immunofluorescent images of HUES-9 NSC passage 22 stained with anti-Sox2, anti-Nestin and DAPI. (G) Immunofluorescent images of HUES-9 NSC passage 22 differentiated for 4 weeks and stained with anti-GFAP and anti-Map2b and DAPI. (H) Same as G, but stained with anti-β-III-tubulin, anti-synapsin and DAPI. (I) Same as G, but stained with anti-GABA and DAPI. (J) Intracellular FACS analysis of hiPSC NDC3.1 sorted NSC passage 16 differentiated for 4 weeks with Nestin, Sox2, DCX and Ki-67. (K) Immunofluorescent images of clonally derived NSC from hiPSC NDC3.1 sorted NSC with anti-human nuclear antigen (hNA), anti-Sox2 and DAPI. (L) Clonally derived NDC3.1 NSC were differentiated for 3 weeks and stained with anti-hNA, anti-GFAP and DAPI. White arrows indicate human astrocytes evidenced by colocalization with hNA. White arrowhead indicates mouse astrocytes that are large and appear more differentiated. (M) An enlargement of the inset from l showing the GFAP+/hNA+ cells. (N) Same as L, but stained with anti-hNA, anti-β-III-tubulin and DAPI. Scale bar is 50 µm.
(TIF)

Figure S5 **Culturing and viability of sorted neurons.** (A) Bright field images of sorted CD184^−^/CD44^−^/CD15^LOW^/CD24^+^ H9 neurons at day 0, 4 and 8 post-FACS. (B) Viability measurements of sorted neurons generated from H9 with the SFEB method. On Day 0, neurons were sorted and counted before plating and 6 hours after plating. Percent survival is measured by the number of cells recovered divided by the number of cells plated. Subsequent time points were taken as indicated. (C) Viability measurements of sorted neurons generated from HUES-9 with the SDIA PA6 co-culture method. Neurons were sorted on Day 0 and counted before plating and 4 hours after plating. Time points were taken as indicated. (D) CD184^−^/CD44^−^/CD15^LOW^/CD24^+^ H9 neurons were stained with TH and DAPI. (E) CD184^−^/CD44^−^/CD15^LOW^/CD24^+^ hiPSC NDC3.1 neurons were stained with GABA and DAPI. (F) Same as D, but stained with ant-Map2b, anti-synaptophysin and DAPI. (G) H9 sorted neurons were co-cultured with human astrocytes for 14 days post-FACS and stained with anti-β-III tubulin, anti-Map2b, anti-GFAP and DAPI. (H) H9 sorted neurons were co-cultured with mouse astrocytes for 7 days post-FACS and stained with anti-β-III tubulin, anti-GFAP and DAPI. (I) HUES-9 sorted CD184^+^/CD44^+^ glia cultured in neuron differentiation medium 7 days post-FACS were stained with anti-β-III tubulin, anti-Nestin, anti-GFAP and DAPI. Scale bar is 50 µm.
(TIF)

Table S1 **Antibodies used for imaging and FACS.** * The antibody panel used was comprised of 189 antibodies to cell surface markers and was the prototype of the product listed. ** Antibodies not commercially available at the time of publication. *** Kind gift from William Stallcup, Sanford-Burnham Medical Research Institute.
(TIF)
