# Supplementary figures and images for: Cell-Surface Marker Signatures for the Isolation of Neural Stem Cells, Glia and Neurons Derived from Human Pluripotent Stem Cells
Source: PLoS One. 2011 Mar 2;6(3):e17540. doi: 10.1371/journal.pone.0017540 (PMC3047583; doi:10.1371/journal.pone.0017540)

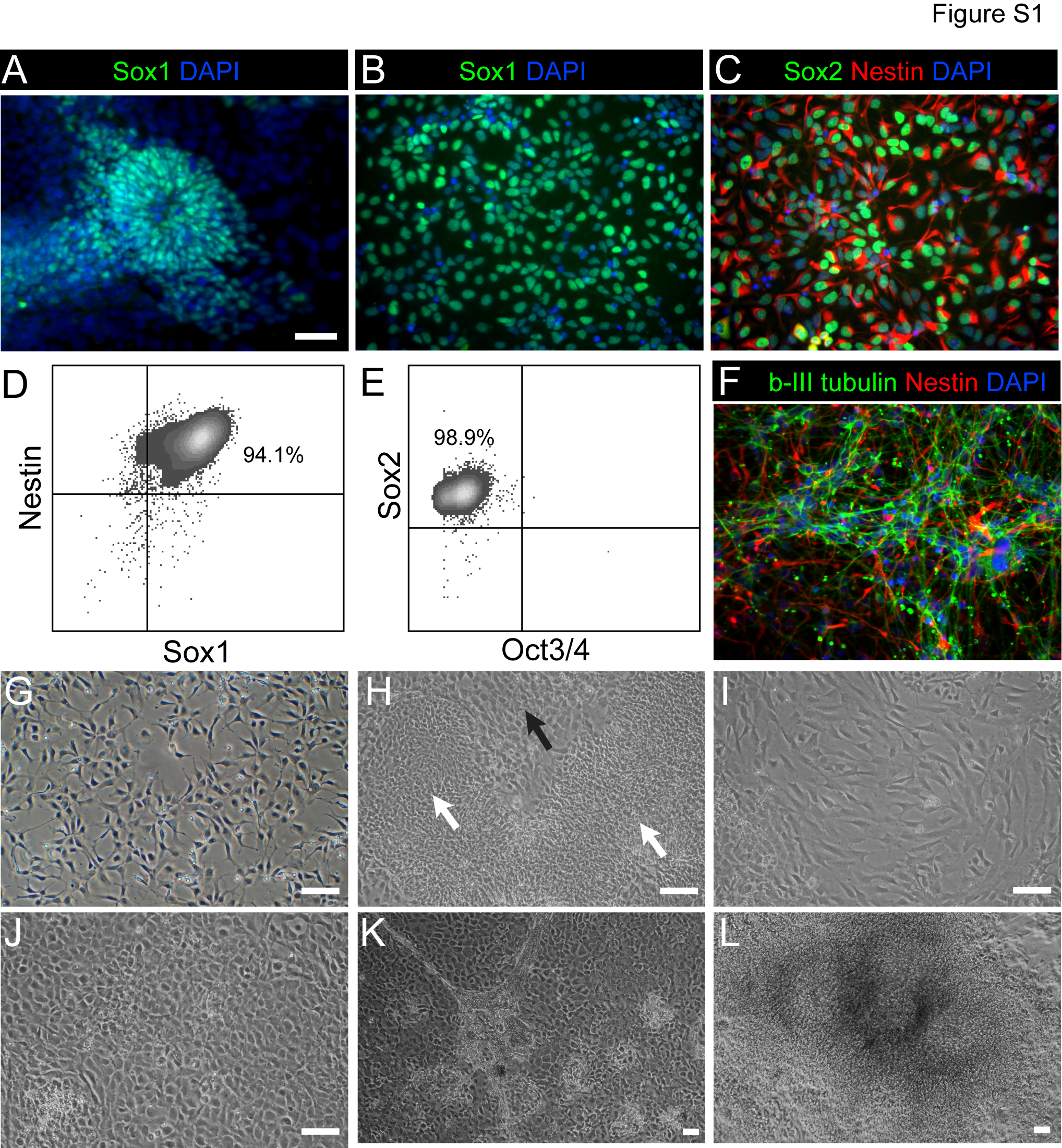

Supplement: Figure S1 — Characterization of cells generated from SFEB culture of H9. (A) Rosette at the EB-rosette(+) stage stained with anti-Sox1 and DAPI. (B) Picked NSC expanded from EB-rosette(+) were stained with anti-Sox1 and DAPI. (C) Same as c but stained with anti-Sox2, anti-Nestin, and DAPI. (D, E) Four-color intracellular FACS analysis with anti-Sox1, anti-Nestin, anti-Sox2 and anti-Oct3/4 of NSC expanded from EB-rosette(+). (F) NSC induced to differentiate for 3 weeks and stained with anti-Nestin, anti-β-III tubulin and DAPI. (G-L) Bright field images of (G) high quality NSC, (H) NSC that are crowded by contaminants (white arrows = NSC, black arrow = contaminants), (I) contaminants at low density, (J) contaminants at high density, (K) EB-rosette(-) depleted of NSC that have been plated and allowed to expand and (L) EB-rosette(+) illustrating columnar rosette structures. Scale bar is 50µm for A-F, 100 µm for G-L. (TIF) [file pone.0017540.s001.tif]

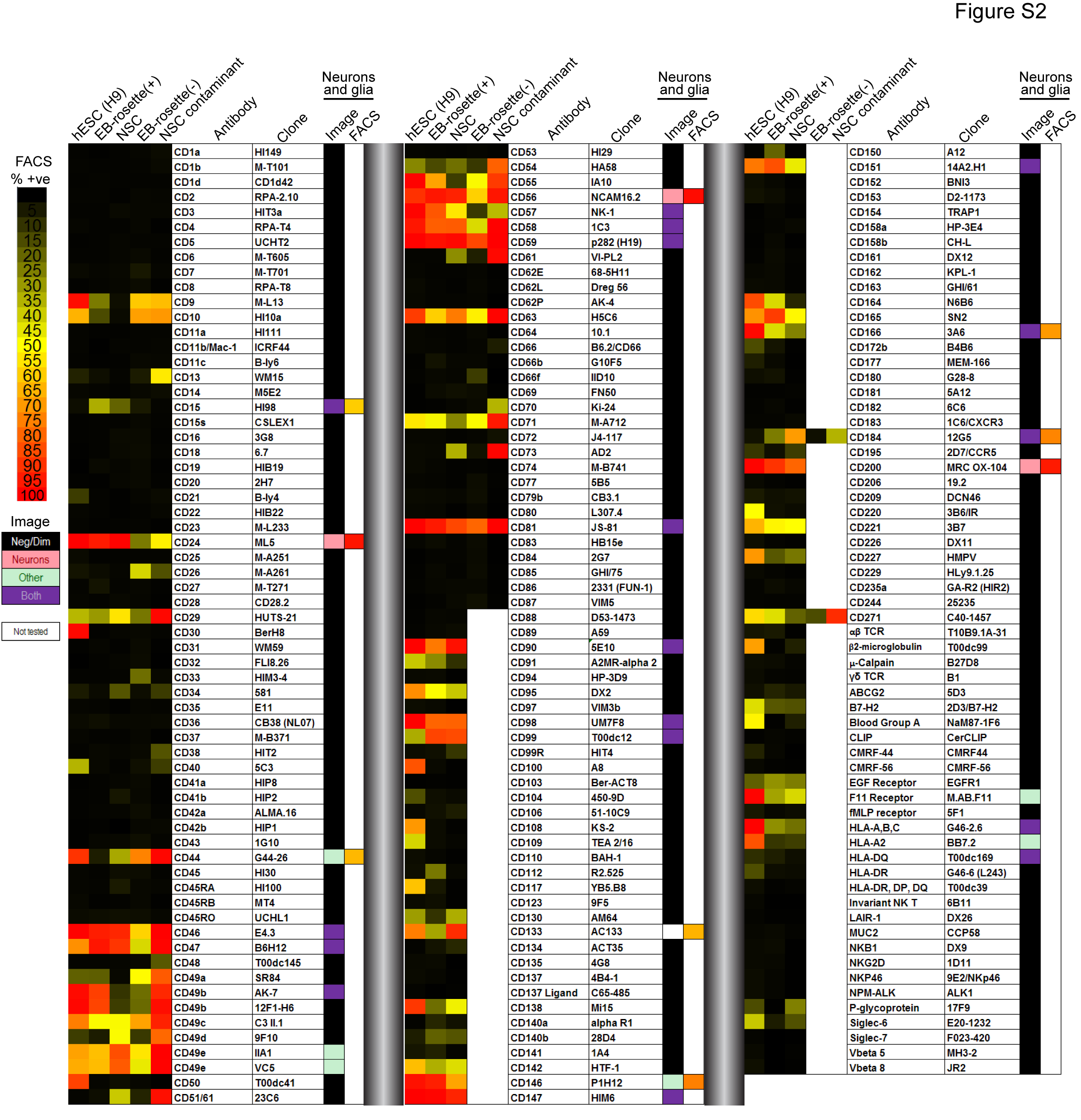

Supplement: Figure S2 — Heat map depicting the results from the FACS and image screens as well as subsequent verification of imaging hits by FACS. The data are organized as percent positive (% +ve) for FACS. Antibody specificity and clone names separate the FACS and image data. EB-rosette(+) = EB with rosettes; EB-rosette(-) = EB depleted of rosettes; NSC = neuronal stem cell expanded from manually isolated EB-rosette(+). NSC contaminant = culture of intermittent contaminant of handpicked and expanded NSC cultures; Neurons and glia = NSC that have been differentiated for 3 weeks and are composed of mixed cultures of neurons, glia and undifferentiated NSC. Images were classified as negative or too dim to determine (Neg/dim) or apparent expression in neurons (pink), non-neuronal cells (Other, blue), or both neurons and non-neuronal cells (Both, purple). Selected hits from the imaging screen were analyzed by FACS. (TIF) [file pone.0017540.s002.tif]

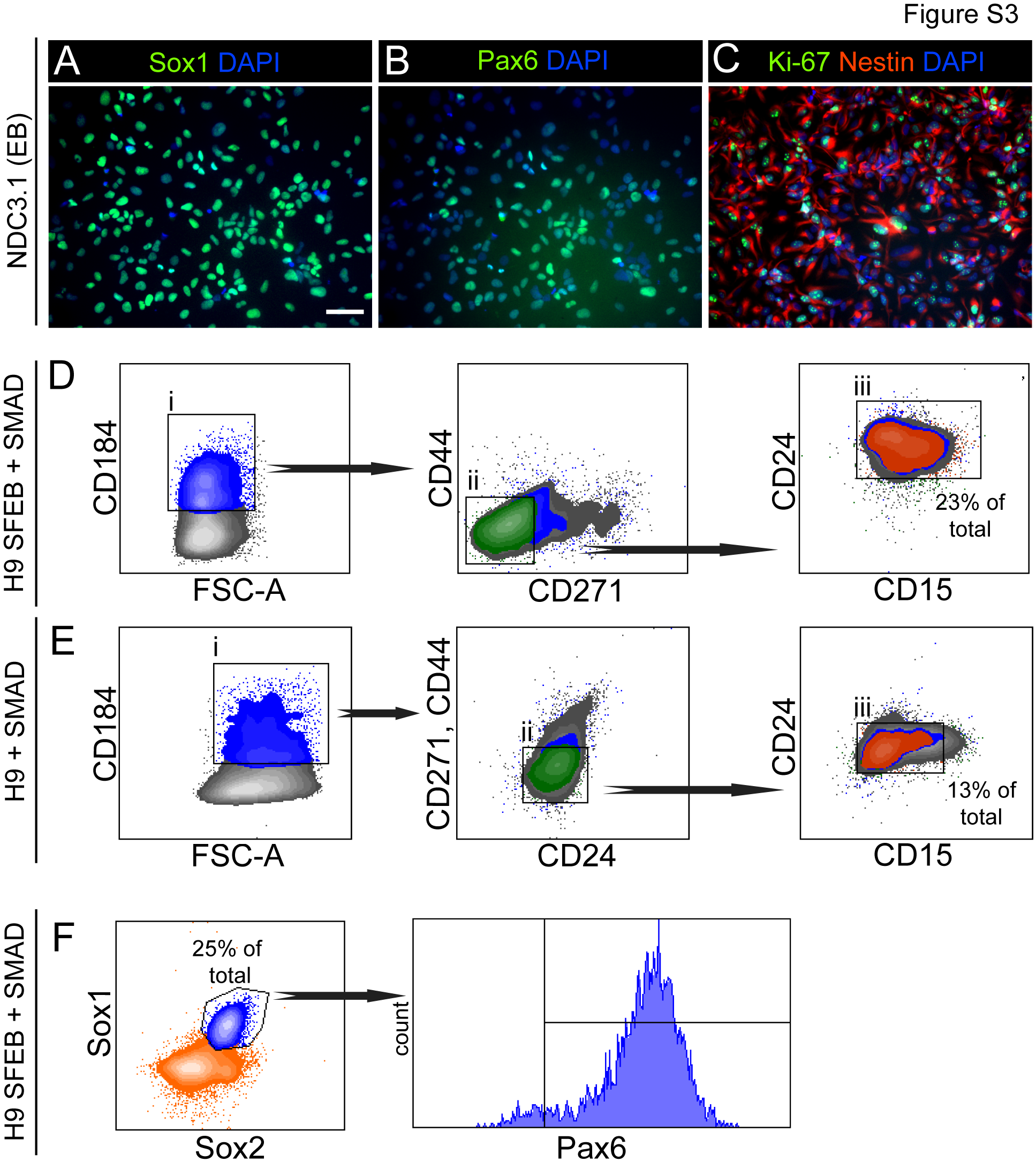

Supplement: Figure S3 — FACS and image data of sorted cells from different neural induction methods. (A, B) Staining with anti-Sox1, anti-Pax6 and DAPI of CD184+/CD271−/CD44−/CD24+ NDC3.1 NSC from PA6 co-culture at the 4th passage after the sort. (C) Same as (a and b) but stained with anti-Ki-67, anti-Nestin, and DAPI. Scale bar is 50 µm. (D) Sorting of H9 after SMAD inhibition with SFEB method. Note that the percentage of likely NSC increases from 10 to 23%. Also CD44 + contaminants are reduced. (E) Sorting of H9 after SMAD inhibition of cells as a monolayer. (F) H9 SFEB cultures that were also treated with dual SMAD inhibition were stained for CD184+/CD271−/CD44−/CD24+ and the cells not selected by the signature were sorted and analyzed for Sox1, Sox2 and Pax6 by intracellular FACS. The two dimensional plot indicates the presence of Sox2+/Sox1+ cells (blue, 24.1% of total). The histogram demonstrates that the Sox2+/Sox1+ cells are also positive for Pax6. (TIF) [file pone.0017540.s003.tif]

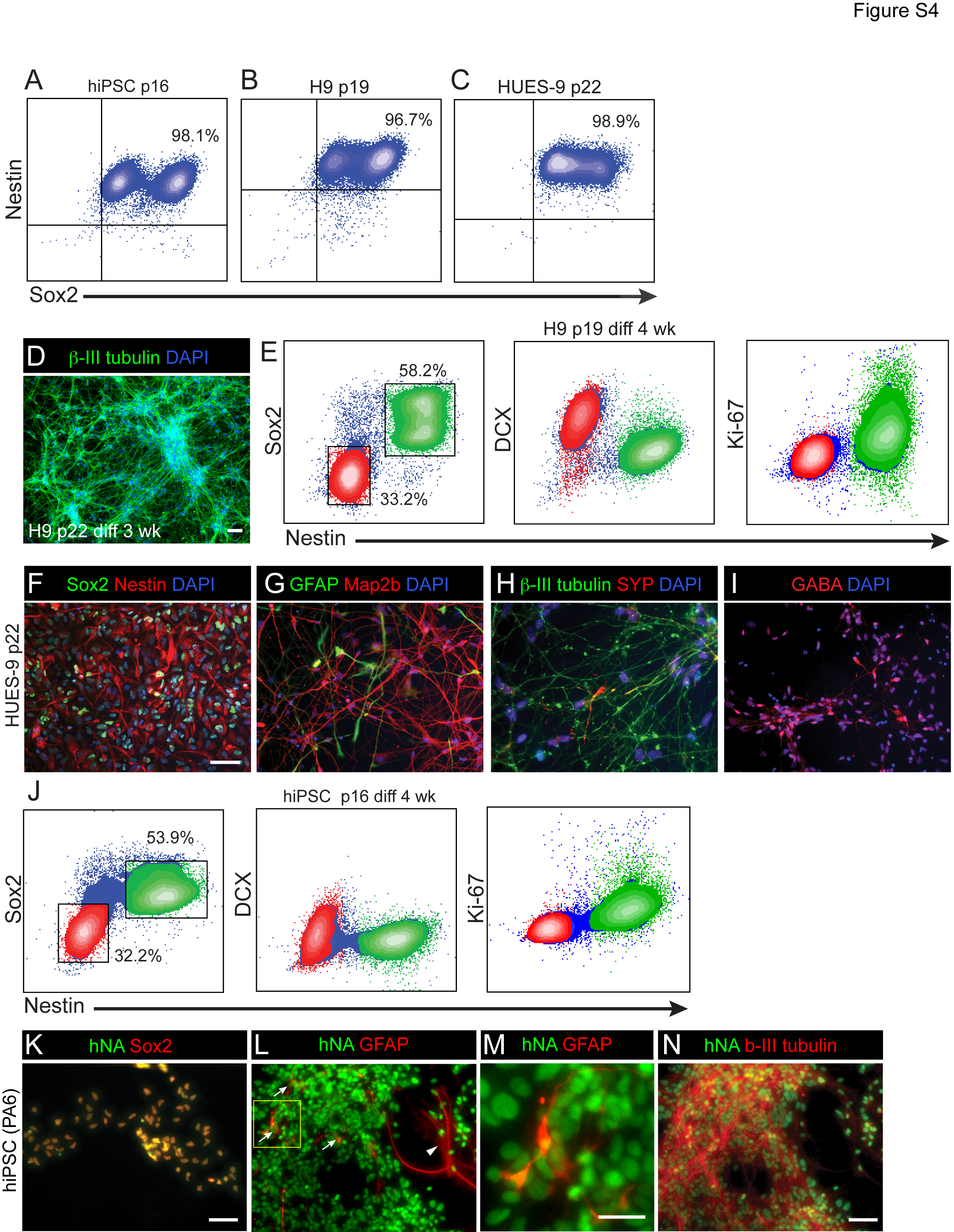

Supplement: Figure S4 — Characterization of long-term NSC cultures. (A-C) Intracellular FACS analysis of NSC cultures: (A) hiPSC NDC3.1 NSC at passage 16, (B) H9 at passage 19 and (C) HUES-9 at passage 22. For these studies we used a new Sox2 antibody that revealed two distinct Sox2 populations. (D) Immunofluorescent image of H9 sorted NSC passage 22 differentiated for 3 weeks and stained with β-III tubulin and DAPI. (E) Intracellular FACS analysis of H9 sorted NSC passage 19 differentiated for 4 weeks with Nestin, Sox2, DCX and Ki-67. (F) Immunofluorescent images of HUES-9 NSC passage 22 stained with anti-Sox2, anti-Nestin and DAPI. (G) Immunofluorescent images of HUES-9 NSC passage 22 differentiated for 4 weeks and stained with anti-GFAP and anti-Map2b and DAPI. (H) Same as G, but stained with anti-β-III-tubulin, anti-synapsin and DAPI. (I) Same as G, but stained with anti-GABA and DAPI. (J) Intracellular FACS analysis of hiPSC NDC3.1 sorted NSC passage 16 differentiated for 4 weeks with Nestin, Sox2, DCX and Ki-67. (K) Immunofluorescent images of clonally derived NSC from hiPSC NDC3.1 sorted NSC with anti-human nuclear antigen (hNA), anti-Sox2 and DAPI. (L) Clonally derived NDC3.1 NSC were differentiated for 3 weeks and stained with anti-hNA, anti-GFAP and DAPI. White arrows indicate human astrocytes evidenced by colocalization with hNA. White arrowhead indicates mouse astrocytes that are large and appear more differentiated. (M) An enlargement of the inset from l showing the GFAP+/hNA+ cells. (N) Same as L, but stained with anti-hNA, anti-β-III-tubulin and DAPI. Scale bar is 50 µm. (TIF) [file pone.0017540.s004.tif]

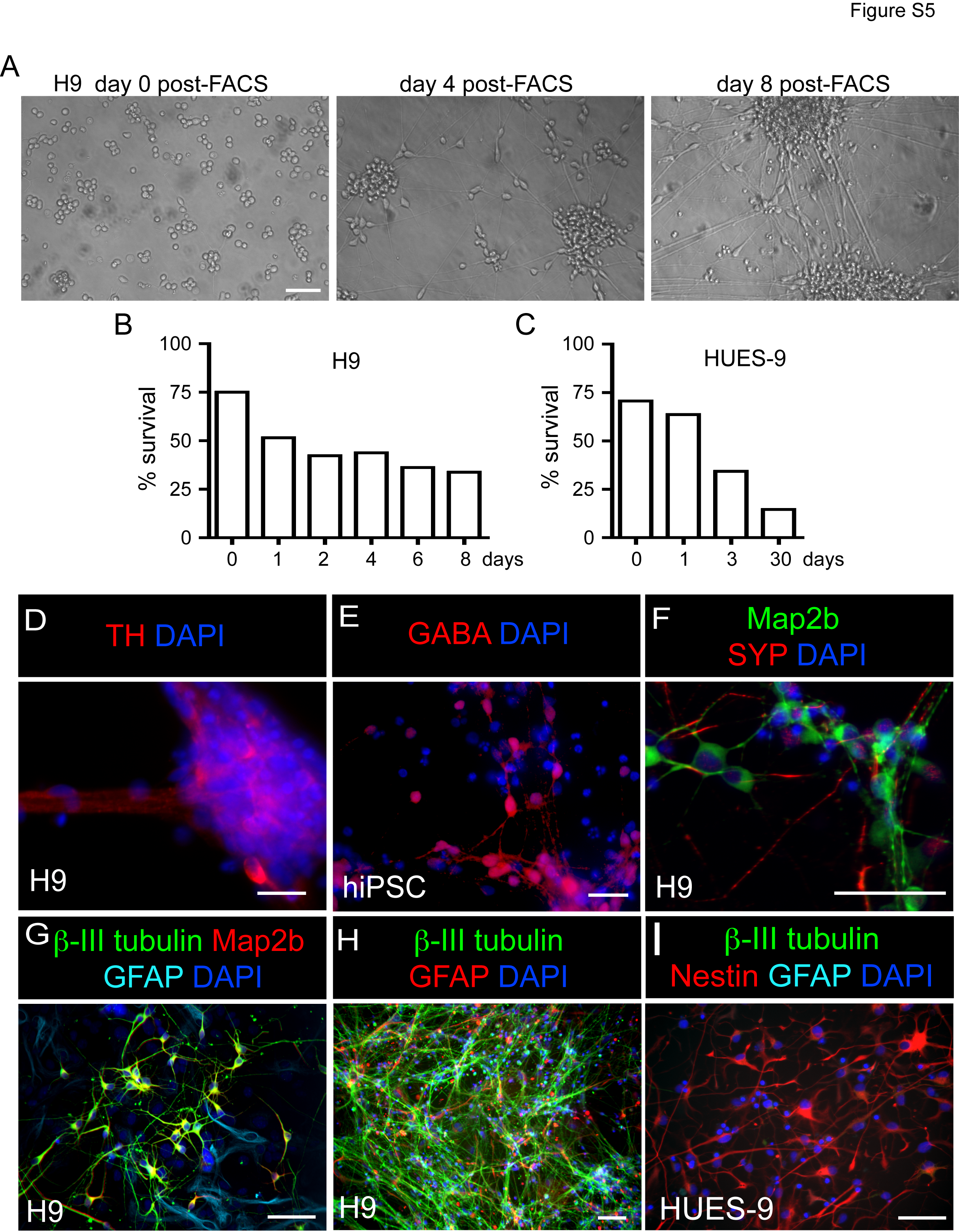

Supplement: Figure S5 — Culturing and viability of sorted neurons. (A) Bright field images of sorted CD184−/CD44−/CD15LOW/CD24+ H9 neurons at day 0, 4 and 8 post-FACS. (B) Viability measurements of sorted neurons generated from H9 with the SFEB method. On Day 0, neurons were sorted and counted before plating and 6 hours after plating. Percent survival is measured by the number of cells recovered divided by the number of cells plated. Subsequent time points were taken as indicated. (C) Viability measurements of sorted neurons generated from HUES-9 with the SDIA PA6 co-culture method. Neurons were sorted on Day 0 and counted before plating and 4 hours after plating. Time points were taken as indicated. (D) CD184−/CD44−/CD15LOW/CD24+ H9 neurons were stained with TH and DAPI. (E) CD184−/CD44−/CD15LOW/CD24+ hiPSC NDC3.1 neurons were stained with GABA and DAPI. (F) Same as D, but stained with ant-Map2b, anti-synaptophysin and DAPI. (G) H9 sorted neurons were co-cultured with human astrocytes for 14 days post-FACS and stained with anti-β-III tubulin, anti-Map2b, anti-GFAP and DAPI. (H) H9 sorted neurons were co-cultured with mouse astrocytes for 7 days post-FACS and stained with anti-β-III tubulin, anti-GFAP and DAPI. (I) HUES-9 sorted CD184+/CD44+ glia cultured in neuron differentiation medium 7 days post-FACS were stained with anti-β-III tubulin, anti-Nestin, anti-GFAP and DAPI. Scale bar is 50 µm. (TIF) [file pone.0017540.s005.tif]

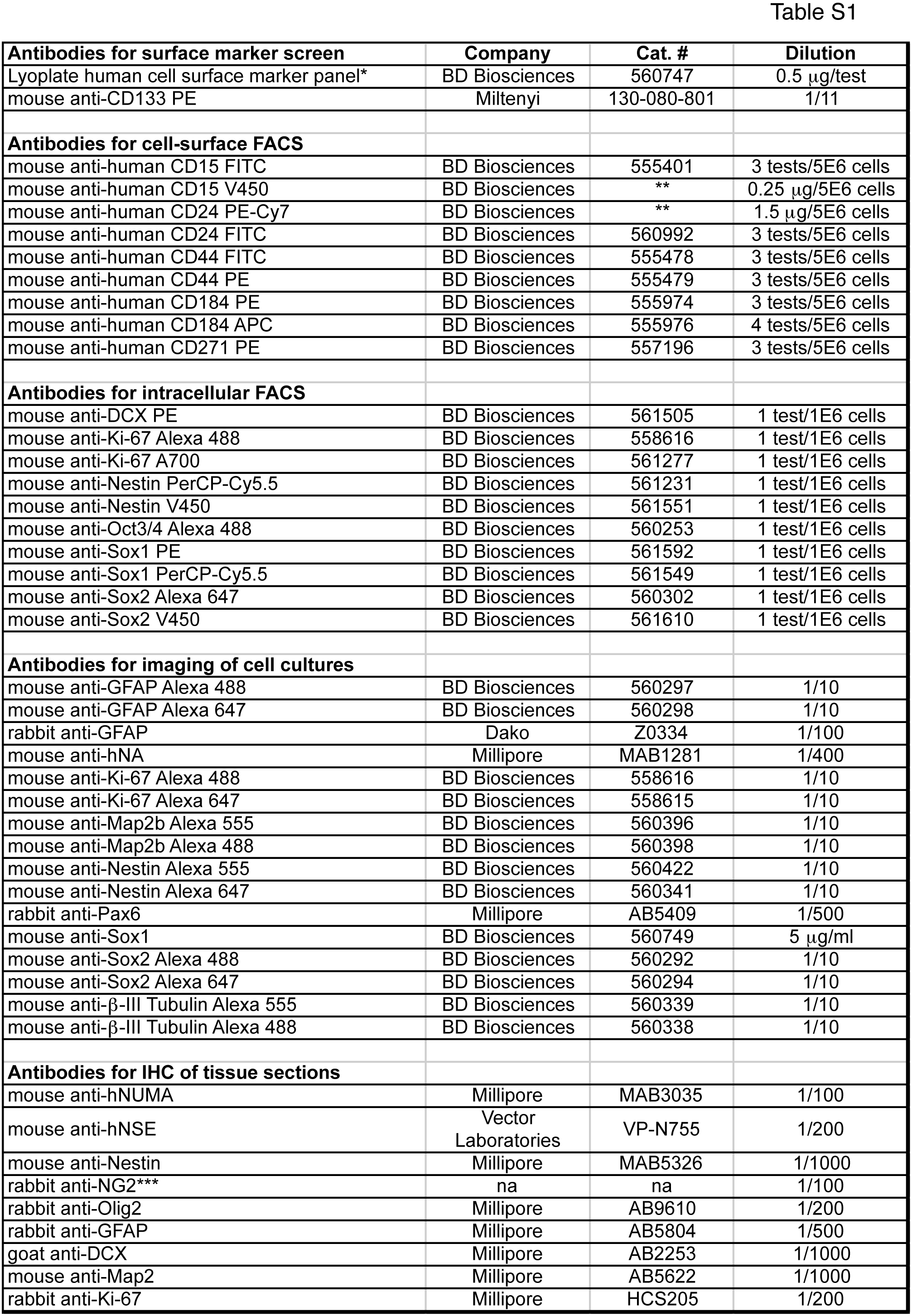

Supplement: Table S1 — Antibodies used for imaging and FACS. * The antibody panel used was comprised of 189 antibodies to cell surface markers and was the prototype of the product listed. ** Antibodies not commercially available at the time of publication. *** Kind gift from William Stallcup, Sanford-Burnham Medical Research Institute. (TIF) [file pone.0017540.s006.tif]
